# The effect of a Life Care Specialist on pain management and opioid-related outcomes among patients with orthopedic trauma: study protocol for a randomized controlled trial

**DOI:** 10.1186/s13063-021-05841-1

**Published:** 2021-11-27

**Authors:** Nicholas A. Giordano, Jesse Seilern und Aspang, J‘Lynn Baker, Cammie Wolf Rice, Bailey Barrell, Lauren Kirk, Erika Ortega, Michelle Wallace, Alaina Steck, Mara L. Schenker

**Affiliations:** 1 Emory University, Nell Hodgson Woodruff School of Nursing, 1520 Clifton Road, Atlanta, GA 30322 USA; 2 Emory University Orthopaedic Surgery, Grady Memorial Hospital, 80 Jesse Hill Jr Drive SE, Atlanta, GA 30303 USA; 3 Christopher Wolf Crusade, Atlanta, GA USA; 4 Grady Memorial Hospital, Atlanta, GA USA; 5 Emory University Department of Emergency Medicine, Grady Memorial Hospital, 80 Jesse Hill Jr Drive SE, Atlanta, GA 30303 USA

**Keywords:** Orthopedic trauma, Life Care Specialist, Opioid utilization, Pain management, Opioid use disorder, Substance misuse, Opioid epidemic, Patient-reported outcomes

## Abstract

**Background:**

Orthopedic trauma patients face complex pain management needs and are frequently prescribed opioids, leaving them at-risk for prolonged opioid use. To date, post-trauma pain management research has placed little emphasis on individualized risk assessments for misuse and systematically implementing non-pharmacologic pain management strategies. Therefore, a community-academic partnership was formed to design a novel position in the healthcare field (Life Care Specialist (LCS)), who will educate patients on the risks of opioids, tapering usage, safe disposal practices, and harm reduction strategies. In addition, the LCS teaches patients behavior-based strategies for pain management, utilizing well-described techniques for coping and resilience. This study aims to determine the effects of LCS intervention on opioid utilization, pain control, and patient satisfaction in the aftermath of orthopedic trauma.

**Methods:**

In total, 200 orthopedic trauma patients will be randomized to receive an intervention (LCS) or a standard-of-care control at an urban level 1 trauma center. All patients will be assessed with comprehensive social determinants of health and substance use surveys immediately after surgery (baseline). Follow-up assessments will be performed at 2, 6, and 12 weeks postoperatively, and will include pain medication utilization (morphine milligram equivalents), pain scores, and other substance use. In addition, overall patient wellness will be evaluated with objective actigraphy measures and patient-reported outcomes. Finally, a survey of patient understanding of risks of opioid use and misuse will be collected, to assess the influence of LCS opioid education.

**Discussion:**

There is limited data on the role of individualized, multimodal, non-pharmacologic, behavioral-based pain management intervention in opioid-related risk-mitigation in high-risk populations, including the orthopedic trauma patients. The findings from this randomized controlled trial will provide scientific and clinical evidence on the efficacy and feasibility of the LCS intervention. Moreover, the final aim will provide early evidence into which patients benefit most from LCS intervention.

**Trial registration:**

ClinicalTrials.govNCT04154384. Registered on 11/6/2019 (last updated on 6/10/2021).

**Supplementary Information:**

The online version contains supplementary material available at 10.1186/s13063-021-05841-1.

## Background

Providing adequate analgesia in the acute orthopedic trauma setting is a critical component of patient care, and opioids currently play a central role. However, opioid prescribing for patients undergoing orthopedic procedures has been identified as a major contributor to the current opioid epidemic. In 2018, opioids were involved in 46,802 overdose deaths nationwide, representing close to 70% of all recorded drug overdoses [1]. The Centers for Disease Control and Prevention estimates that 38 people die each day in the United States from overdoses involving prescription opioids, on average [2]. Still, prescription-originated addiction and opioid misuse, two major constituents of the opioid epidemic, have not seen a decline in incidence since 1999, despite increased legislative oversight in physician-prescribing practices [3].

Patients with orthopedic trauma frequently experience difficulties accessing pain care, despite requiring complex pain medication, care coordination, and substance use counseling [4–6]. This is of concern given that orthopedic trauma patients are particularly vulnerable to develop chronic pain and even substance use disorders [7–9]. The strongest risk factor for developing opioid use disorder is a pre-existing substance use disorder [10]. Trauma patients are the most likely patient category to be under the influence of psychoactive drugs and alcohol use at the time of hospital admission [5]. In addition, trauma patients are more likely to have used prescription opiates prior to admission when compared to the general population [11]. Thus, orthopedic trauma patients are one of the most at-risk patient groups for opioid misuse and abuse [12]. Despite the use of prescription opioids declining over the past decade, often at the expense of effective pain control for patients, orthopedic trauma care pain management continues to be centered around opioids [13, 14].

In the last 15 years, there has been an overwhelming response by funding and legislative agencies that have targeted prescriber practices to curb opioid dispensing [13, 15]. Relatively few patient-oriented approaches have been proposed to mitigate opioid-related risks while simultaneously promoting effective multimodal pain management, and even fewer have shown actual benefit in reducing opioid utilization or risk of opioid-involved overdose. In a prospective randomized controlled trial, McCarthy et al. found that a complex, multifaceted educational effort involving informational handouts, multiple physician/pharmacy reminders, and SMS text prompts improved medication knowledge, yet failed to decrease actual consumption of opioids [16]. However, in this study little to no emphasis was placed on the evaluation of pain. This holds true for the vast majority of research in this field, as interventions are primarily focused on the relationship between medical intervention, social determinants and health and health policy, and opioid medication consumption/dispensing patterns, with a frank underrepresentation of the influence of pain interference and the impact of patients’ pain on opioid consumption [17].

Nevertheless, the findings of McCarthy et al. support the notion that possessing knowledge about medication risks is likely important but is insufficient to ensure safe use, likely because medication-taking behaviors are often influenced by complex factors, including health literacy, self-efficacy, and attitudes [16, 18]. Several additional studies have demonstrated benefit to preoperative education in defining postoperative pain expectations in elective orthopedic surgery [19–23], but there continues to be a paucity of evidence that incorporates personalized risk assessment and targeted education in acute care settings. Moreover, few studies have evaluated alternative non-pharmacologic strategies to cope with pain in the acute care setting following orthopedic trauma.

Hence, in an effort to develop a comprehensive pain management protocol with key stakeholders in the Opioid Epidemic, an academic-community partnership with the Christopher Wolf Crusade (CWC) was established. CWC is a non-profit organization that facilitates prevention, solutions, education, and advocacy for the American Opioid Epidemic [24]. CWC’s primary focus in the acute care setting is to hire, train, and implement a novel member of the healthcare team, a Life Care Specialist (LCS), who focuses on non-pharmacologic, behavior-specific intervention, and personalized opioid education. Their patient-centric approach seeks to decrease overall opioid utilization while also optimizing postoperative pain management by implementing alternative non-pharmacologic strategies. Additionally, LCS implement standardized substance use risk assessments, coordinate inpatient care, and train both patients and caregivers on harm reduction strategies, specifically how to use naloxone, a potential lifesaving opioid reversal agent. This randomized controlled trial aims to (1) evaluate the effectiveness of LCS intervention on pain management and opioid utilization in the acute inpatient trauma setting and immediately after, as participants transition home, and (2) evaluate patient knowledge of the risks of opioid misuse and abuse, compared to the current standard of care for patients in the aftermath of orthopedic trauma at our level 1 trauma center. We hypothesize that the LCS will improve pain control, while minimizing opioid use, and will also encourage safe disposal patterns following successful opioid tapering.

## Methods/design

### Study design

In this single-center randomized, controlled trial (RCT), eligible participants will be randomized to receive either LCS intervention or current standard of care for pain management. Participants are screened for eligibility, consented, enrolled, and administered baseline measures within 24 h of their surgery. After hospital discharge, all participants will be assessed at 2, 6, and 12 weeks for evaluation of outcome measures, including pain management and opioid utilization. The trial has been approved by the Institutional Review Board of Emory University (IRB00115061) (Version 5.0) and has been registered with ClinicalTrials.gov (NCT04154384) on 11/6/2019 (last updated on 6/10/2021). Trial registry data is visualized below (Table 1).

**Table 1:** Trial registry data set

First submitted date	November 2, 2019	
**First posted date**	November 6, 2019	
**Actual study start date**	February 21, 2020	
**Current primary outcome measures (submitted: June 8, 2021)**	• Opioid pain medication usage [time frame: month 12] The primary outcome of the single-arm pilot study will be the utilization of opioid pain medication at 1 year after the operation. This value will be compared to the 23% of a historical control cohort reporting continuing to use opioids a year post operation. • Change in Pain Management Questionnaire (PMQ) Score [time frame: day 1 (during inpatient hospitalization), week 2, week 6, month 3] For participants in the dual-arm, clinical trial portion of the study, the risk of opioid misuse is assessed with the PMQ. The PMQ is a 26-item questionnaire where responses are given on a 5-point Likert scale where 0 = disagree and 4 = agree. Total scores range from 0 to 104 where higher scores indicate increased risk of opioid misuse. • Change in Prescription Drug Use Questionnaire - Patient Version (PDUQp) Score [time frame: day 1 (during inpatient hospitalization), week 2, week 6, month 3] For participants in the dual-arm, clinical trial portion of the study, prescription drug use is assessed with the self-report version of the Prescription Drug Use Questionnaire. The PDUQp includes 31 items which are responded to as “yes” or “no.” Responses of “yes” are coded as 1. Only 30 items are summed to provide a total score which can range from 0 to 30. Higher responses indicate opioid misuse behaviors. • Change in Patient-Reported Outcomes Measurement Information System (PROMIS) Sleep Disturbance - Short Form Score [time frame: day 1 (during inpatient hospitalization), week 2, week 6, month 3] For participants in the dual-arm, clinical trial portion of the study, severity of insomnia, sleep disruption, and sleep quality over the past 7 days is assessed with the 4-item PROMIS Sleep Disturbance - Short Form. Responses are given on a 5-point Likert scale where 1 is equivalent to best possible and 5 is equivalent to worst possible. Raw scores are converted to t-scores ranging from 0 to 100, with a mean of 50 and standard deviation of 10. Scores below 50 indicate better sleep than the average person. • Change in PROMIS Physical Function - Short Form Score [time frame: day 1 (during inpatient hospitalization), week 2, week 6, month 3] For participants in the dual-arm, clinical trial portion of the study, self-reported capability to conduct physical activity is assessed with the PROMIS Physical Function - Short Form. Responses to the 4 items are given on a 5-point Likert scale where 1 = unable to do and 5 = without any difficulty. Raw scores are converted to t-scores ranging from 0 to 100, with a mean of 50 and standard deviation of 10. Scores above 50 indicate better physical function than the average person. • Change in PROMIS Pain Interference - Short Form Score [time frame: day 1 (during inpatient hospitalization), week 2, week 6, month 3] For participants in the dual-arm, clinical trial portion of the study, the extent to which pain has impeded engagement with social, cognitive, emotional, physical, and recreational activities over the past 7 days is assessed with the PROMIS Pain Interference - Short Form. Responses to the 4 items are given on a 5-point Likert scale where 1 = not at all and 5 = very much. Raw scores are converted to t-scores ranging from 0 to 100, with a mean of 50 and standard deviation of 10. Scores below 50 indicate less pain interference than the average person. • Change in Opioid Literacy Tool (OLT) Score [time frame: day 1 (during inpatient hospitalization), month 3] For participants in the dual-arm, clinical trial portion of the study, accuracy of knowledge about opioids (3 questions) and opioid-related risks (5 questions) is assessed with an OLT. Accuracy of opioid knowledge responses are given on a dichotomous scale (yes/no). Responses for accuracy of knowledge about opioid-related risks are given on a 7-point scale where 1 = definitely true and 7 = definitely false. For these 5 questions, total scores range from 5 to 35 and higher scores indicate improved literacy (accurate understanding of prescription opioid addiction-risk, opioid dependence, and risk of opioid overdose). • Change in Primary Care Posttraumatic Stress Disorder Screen (PC-PTSD-5) Score [time frame: day 1, month 3] For participants in the dual-arm, clinical trial portion of the study, the primary care post-traumatic stress disorder (PTSD) screen for the Diagnostic and Statistical Manual of Mental Disorders (DSM-5; PC-PTSD-5) will be administered. The PC-PTSD-5 is a 5-item instrument used to assess previous exposure to traumatic events and subsequent presence of the DSM-5 diagnostic criteria for PTSD. Responses are given as “yes” or “no” and each response of “yes” is scored as one point. Total scores range from 0 to 5 where higher scores indicate greater symptoms of PTSD. In primary care settings, a minimum of 3 points is considered probable PTSD.	
**Original primary outcome measures (submitted: November 5, 2019)**	• Opioid pain medication usage [time frame: month 12] The primary outcome of the study will be the utilization of opioid pain medication at 1 year after the operation. This value will be compared to the 23% of a historical control cohort reporting continuing to use opioids a year post operation.	
**Current secondary outcome measures (submitted: June 8, 2021)**	• Change in numeric rating scale average pain score [time frame: day 1 (during inpatient hospitalization) up to discharge (until week 2), week 6, month 3] For participants in the single-arm pilot study and in the dual-arm, clinical-trial portion of the study, daily pain within the last 24 h will be assessed using a 10-point Likert scale where 1 = no pain and 10 = severe pain. After week 2, pain will be assessed only during the follow-up visits. • Change in opioid utilization [time frame: day 1 (during inpatient hospitalization) up to discharge (until week 2), week 6, month 3] For participants in the single-arm pilot study, and in the dual-arm, clinical-trial portion of the study, opioid utilization will be recorded in daily morphine milligram equivalents. After week 2, opioid utilization will be assessed only during the follow-up visits. • Change in average steps per day [time frame: day 1 (during inpatient hospitalization) up to discharge (until week 2)] For participants in the dual-arm, clinical trial portion of the study, wrist-actigraphy devices will capture continuous postoperative functional outcomes among patients during their hospitalization and up to 2-weeks postoperatively. Activity will be measured as the average number of steps per day. • Change in total sleep time [time frame: day 1 (during inpatient hospitalization) up to discharge (until week 2)] For participants in the dual-arm, clinical trial portion of the study, wrist-actigraphy devices will capture continuous postoperative functional outcomes among patients during their hospitalization and up to 2 weeks postoperatively. Total sleep time is assessed in minutes of sleep per night. • Change in sleep latency [time frame: day 1 (during inpatient hospitalization) up to discharge (until week 2)] For participants in the dual-arm, clinical trial portion of the study, wrist-actigraphy devices will capture continuous postoperative functional outcomes among patients during their hospitalization and up to 2 weeks postoperatively. Sleep onset latency is assessed as the length of time, in minutes, that it takes to transition from wakefulness to sleep. • Change in sleep fragmentation [time frame: day 1 (during inpatient hospitalization) up to discharge (until week 2)] For participants in the dual-arm, clinical trial portion of the study, wrist-actigraphy devices will capture continuous postoperative functional outcomes among patients during their hospitalization and up to 2 weeks postoperatively. Sleep fragmentation is assessed as the number of awakenings and sleep stage shifts divided by sleep time. • Change in wake after sleep onset [time frame: day 1 (during inpatient hospitalization) up to discharge (until week 2)] For participants in the dual-arm, clinical trial portion of the study, wrist-actigraphy devices will capture continuous postoperative functional outcomes among patients during their hospitalization and up to 2 weeks postoperatively. Wake after sleep onset is assessed as the periods of wakefulness occurring after sleep onset. • Change in sleep efficiency [time frame: day 1 (during inpatient hospitalization) up to discharge (until week 2)] For participants in the dual-arm, clinical trial portion of the study, wrist-actigraphy devices will capture continuous postoperative functional outcomes among patients during their hospitalization and up to 2 weeks postoperatively. Sleep efficiency is the percentage of time in bed spent sleeping (total sleep time/sleep period time × 100). • Patient satisfaction survey [time frame: week 2] For participants in the single-arm pilot study and in the dual-arm, clinical-trial portion of the study, patient satisfaction with clinical care will be assessed with a modified Press Ganey Integrated Survey. Integrated study-specific questions will align with the conventional rating scale of “strongly agree”–“strongly disagree.” This survey will capture a comprehensive picture of each participant's care experience. Higher scores indicate higher satisfaction and will be compared among study arms and to the Hospital Consumer Assessment of Healthcare Providers and Systems (HCAHPS) comparative feedback database.	
**Original secondary outcome measures (submitted: November 5, 2019)**	• Change in pain score [time frame: week 2, week 6, month 3, month 6, month 12] Pain will be assessed using an 11-point Likert scale where 0 = no pain and 10 = severe pain. • Change in opioid utilization [time frame: day 1 (at hospital discharge), week 2, week 6] Opioid utilization will be recorded in morphine equivalents.	
**Brief title**	**Life** Care **Specialists** (LCS) with a focus on patient **pain** management and prevention of substance misuse	
**Official title**	**Life** Care **Specialists** (LCS) with a focus on patient **pain** management and prevention of substance misuse	
**Study type**	Interventional	
**Study phase**	Not applicable	
**Study design**	Allocation: Randomized Intervention model: Parallel assignment Intervention model description: There were 121 participants in the single-arm pilot trial of this study where the intervention was refined. The clinical trial portion of this study randomizes participants to receive the intervention or the standard of care. Masking: None (open label) Primary purpose: Prevention	
**Condition**	Opioid use	
**Intervention**	• Behavioral: **Pain** management strategies The **Life** Care **Specialist** (LCS) teaches evidence-informed behavioral interventions and will work with the patient to develop personalized **pain** management strategies focused on behavioral education, including progressive muscle relaxation (PMR), the Community Resiliency Model (CRM®), motivational interviewing, and reflective listening. • Behavioral: **Life** Care **Specialist** (LCS) Intervention The **Life** Care **Specialist** (LCS) uses a two-arm approach to education by initially assessing participants general understanding of opioids upon which targeted education is tailored and applied and secondly, building a longitudinal relationship with each patient to increase the saliency of administered opioid education during postoperative follow-up. Information includes proper disposal, common symptoms of opioid use, signs of dependence and overdose and use of naloxone. Information is disseminated orally with adjunct physical resource guides including visual representations and literature. Other name: Opioid education • Other: Clinical coordination with referrals The **Life** Care **Specialist** (LCS) can help arrange a referral for the participant, should a medical or social issue be identified during LCS intervention, including mental health services, addiction medicine services, housing insecurity referrals, food insecurity referrals, and amputee support. When giving referrals, the LCS works closely with physicians and nurses to make sure that the participant is a good fit for the referral program.	
**Study arms**	• Experimental: Pilot study of **pain** management strategies Orthopedic trauma patients will work with a **Life** Care **Specialist** (LCS) and will receive personalized **pain** management strategies to avoid potential opioid misuse. Participants will be followed for 1 year post operation. An official **pain** management protocol will be developed during the pilot portion of this study. Intervention: Behavioral: **Pain** Management Strategies • Experimental: **Life** Care **Specialist** (LCS) Intervention In addition to receiving current standard-of-care for **pain** management in the aftermath of trauma, participants will have the full communication of opioid risk—via the validated Opioid Risk Tool (ORT) and a detailed substance abuse and mental health screening. As part of the daily LCS intervention, the inpatients will engage in behavioral **pain** management, opioid education and harm-reduction strategies (naloxone education), while also being screened for eligibility for respective referrals for complex needs, such as mental health and substance use disorders. Upon discharge, each participant will be educated by the LCS on future available modes of contact (telephone, email, video-call, follow-up visits at 2, 6, and 12 weeks). Intervention: Behavioral: **Life** Care **Specialist** (LCS) Intervention • Active comparator: Standard of care with clinical coordination Participants will receive the current standard-of-care for **pain** management in the aftermath of trauma, including a standardized prescription protocol, and hospital-system approved discharge instructions which provide written instruction on how to taper opioid use and links to written/online resources for opioid misuse, overdose prevention, and State-approved disposal options. Intervention: Other: Clinical coordination with referrals	
**Recruitment status**	Enrolling by invitation	
**Estimated nrollment (submitted: June 8, 2021)**	321	
**Original estimated enrollment (submitted: November 5, 2019)**	200	
**Estimated study completion date**	January 2022	
**Estimated primary completion date**	January 2022 (Final data collection date for primary outcome measure)	
**Eligibility criteria**	Inclusion criteria for single-arm pilot portion of this study: • Orthopedic trauma patients with planned surgical procedure • Informed consent obtained Exclusion criteria for single-arm pilot portion of this study: • Enrolled in a study that does not permit co-enrollment • Unlikely to comply with the follow-up schedule • Unable to converse, read or write English or Spanish at elementary school level Inclusion criteria for clinical trial portion of this study: • Orthopedic trauma patients with an isolated injury requiring surgery • Informed consent obtained • Functioning cellphone Exclusion criteria for clinical trial portion of this study: • Enrolled in a study that does not permit co-enrollment • Unlikely to comply with the follow-up schedule • Unable to converse, read or write English or Spanish at elementary school level • Unlikely to complete surveys at home, access to phone • Unlikely to respond to opioid utilization text messaging (SMS) • Incarcerated • Pregnant	
**Sex/gender**	Sexes eligible for study: All	
**Ages**	18 years and older (adult, older adult)	
**Accepts healthy volunteers**	No	
**Listed location countries**	United States	
**NCT number**	NCT04154384	
**Other study ID numbers**	IRB00115061	
**IPD sharing statement**	Plan to share IPD:	Yes
	Plan description:	Deidentified, individual participant data will be made available for sharing upon request from other researchers.
	Supporting materials:	Study protocol
	Supporting materials:	Statistical Analysis Plan (SAP)
	Time frame:	Individual participant data will be available for sharing following publication of the findings from this study until 5 years after publication.
	Access criteria:	Researchers interested in accessing data should provide a description of the proposed project to Dr. Schenker at mara.schenker@emory.edu.
**Study sponsor**	Emory University	
**Collaborators**	Christopher Wolf Crusade (CWC)	
**Investigators**	Principal Investigator: Mara Schenker, MD, Emory University	

### Participants

This study will include 200 adult patients (100 per group) who have been admitted to the institution’s trauma center with an acute isolated orthopedic injury. Detailed inclusion and exclusion criteria are listed in Table 2. Final eligibility will be determined by the clinical research coordinator (CRC) and principal investigator. Informed consent will be obtained from the CRC (Additional file 1). Dropout criteria were individuals who were unable to participate, refused to participate or who were incarcerated after surgery. Inclusion criteria were adult male and female patients (18 years or older), who were admitted to the orthopedic trauma service with an isolated orthopedic injury requiring surgery, who are able to give verbal and written informed consent and have a working cell phone for future research related communication. Pregnant, incarcerated, illiterate or non-English speaking patients, and patients who are unlikely to comply with the follow-up schedule, or were already enrolled in a study that does not permit dual-enrollment will be excluded. Recruitment goals will be met by screening all patients who are admitted to the orthopedic trauma service for eligibility criteria daily during the study period.

**Table 2:** Eligibility criteria

Inclusion criteria	Exclusion criteria
• Male and female patients 18 years of age or older • Orthopedic trauma patients with an isolated injury requiring surgery • Informed consent obtained • Working cell phone	• Enrolled in a study that does not permit co-enrollment • Unlikely to comply with the follow-up schedule • Unable to converse, read or write English at elementary school level • Unlikely to complete surveys at home, access to phone • Incarcerated • Pregnant

### Randomization and blinding

A random sequence generation process will be used for this study and implemented by the P.I. (MS). Randomization will occur at the level of the treating orthopedic trauma surgeon, using a computer-based random number generator we will randomize all patients treated by a specific surgeon to either intervention or control arm. The clinical team (orthopedic surgeon and advanced practice provider) and research staff, and statistician will be blinded to the group allocation throughout the study period. The LCS providing the intervention, one clinical research coordinator, and the principal investigator will be the only unblinded staff. Allocation concealment will be upheld as research staff surveys and LCS will schedule patient interactions (research staff collecting patient data from participants vs. LCS intervention) at separate times, limiting the risk of unblinding. Furthermore, the LCS will assign their participants unique ID’s.

### Control Group

Standard-of-care for post-operative pain management in orthopedic trauma patients at our institution includes a pharmacologic protocol that has been adapted for use in our organization from prior published work [25]. On discharge, all participants (intervention and control group) will receive Roxicodone 5 mg every 8 h as needed for the first two post-discharge weeks, with scheduled acetaminophen (1000 mg every 8 h) and ibuprofen (600 mg every 8 h). At the first post-discharge visit (between 2 and 3 weeks post-discharge), participants will receive refills (if needed) for oxycodone/acetaminophen (5 mg/325 mg) every 12 h for 1 week, followed by every 24 h for 1 week (dispense 21 pills). In addition, ibuprofen will be prescribed as needed. After the first post-discharge visit, opioid medications will not standardly be prescribed, and acetaminophen and ibuprofen will be prescribed as needed. Off-protocol refills will be documented in the participants’ notes and will be reported to the Prescription Drug Monitoring Program (PDMP).

In addition, standard instructions will be provided to all participants (intervention and control group) and will be delivered to participants by a nurse educator at hospital discharge (Additional file 1).

### Intervention

The role of the LCS is based on the success seen in introducing paraprofessionals and peer navigators into other clinical practice settings. For example, in pediatric settings, there is significant evidence showing that non-pharmacological interventions delivered by Certified Child Life Specialists lead to more cooperation, reduction in perceived pain, and higher satisfaction scores from patients and their families [26, 27]. Patient navigators have been shown to improve care outcomes for patients with numerous chronic conditions, including human immunodeficiency virus and cancer [28, 29]. Recently, patient navigators have been utilized in emergency department settings to engage patients with substance use disorders in conversations about initiating medication-assisted treatment [30, 31]. Patient coaches, used in outpatient care settings, have been shown to improve chronic pain outcomes [32]. Similar to these other paraprofessional roles, LCS are positioned to improve pain management by coaching participants on nonpharmacological pain management approaches. Uniquely, LCS provide opioid education and coordinate care for participants both during hospitalization and as they transition home.

Our community partners at the Christopher Wolf Crusade train all LCS using a months-long intensive immersive curricula where they shadow surgeons, toxicologists, pain management clinicians, social workers, and harm reduction experts in tandem to participating in didactic course work that focuses on the pathophysiology of substance use and pain. Didactic training is taught by trained faculty through Emory University, as part of its online certificate course work. Trainees also complete an online self-paced Social Determinants of Health course taught by tenured faculty at Emory. All LCS are trained by pain management and trauma care experts at Emory University to provide participants with non-pharmacologic pain management strategies based on the validated Community Resiliency Model (CRM) [33, 34]. Specifically, this evidenced-based model works on a train the trainer approach. Pain management clinicians and harm reduction experts from Emory University and community partners, such as the Atlanta Harm Reduction Coalition, provide LCS the skills to teach patients appropriate non-pharmacologic pain management interventions.

The LCS is introduced to the participant postoperatively, after study enrollment. The LCS will have access to the initial survey data, including individualized risk of opioid misuse (Opioid Risk Tool (ORT)), prior substance use history, depression screening, as well as a full assessment of social determinants of health. The LCS uses these data to inform and personalize their intervention.

There are three main prongs to the LCS intervention.

1. Behavioral pain management strategies
2. Opioid education
3. Coordination of care and referrals

### Behavioral pain management

The LCS teaches evidence-informed behavioral interventions: Progressive muscle relaxation (PMR), the Community Resiliency Model (CRM®) [35], motivational interviewing, and reflective listening.

PMR is used to help participants relax the body and is a technique commonly used for promoting the release of muscular tension [36]. PMR involves focusing attention on different muscle groups. LCS begin with the feet and move up the body to the face. Participants tense muscles and then relax them (for example, tightening the abdomen and then releasing it). LCS have participants tense and release each muscle group three times for the optimal effect. Research suggests that PMR can activate the production of natural opiates and promote the optimal function of the immune system [37]. These suggested benefits directly relate to the pain management goal of the LCS.

CRM is a model for well-being that was developed by the Trauma Resource Institute. The model is an evidence-based intervention designed to help individuals who have experienced highly stressful and traumatic events. It is based on cutting-edge neuroscience including concepts of neuroplasticity, neurogenesis, and interoceptive awareness. The LCS educates participants on common trauma reactions and the ways that the autonomic nervous system reacts. The CRM consists of six skills that can be used at different levels of literacy and with minimal effort or supplies by the participant. The skills have been used with frontline healthcare workers, with female addiction patients, and in crisis situations [38, 39].

Additionally, LCS are trained in motivational interviewing and reflective listening. These skills shape discussions with participants about their injuries in a trauma and resiliency-informed manner. Motivational interviewing is a technique, process, or style that enables the LCS to interact with participants in a nonjudgmental way that helps them resolve the ambivalence that prevents them from realizing personal goals [40]. The overall goal is to enhance the participant’s readiness to change. It does not operate from a deficiency model that seeks to instill knowledge, insight, skills, correct thinking, or even motivation. Rather, the helping professional seeks to evoke the participant’s own motivation, with confidence in the human desire and capacity to grow in positive directions. Reflective Listening is a strategy of listening to others with respect, compassion, and attention. The underlying goal is to understand what the other is saying from their perspective.

### Opioid Education

The secondary focus of the LCS intervention is opioid education. Current literature on inpatient opioid education suggests that it is inadequate [41]. The LCS intervention, however, uses a two-arm approach to education. The LCS first assesses participants on their general understanding of opioids during the pre-intervention Opioid Literacy Tool (OLT) and second, the LCS builds a longitudinal trust relationship with the participant.

The pre-intervention questionnaire (OLT) allows the LCS to target specific areas of understanding for each participant. For example, a participant who does not know what an opioid is needs complete education, while a participant who knows what opioids are and understands the risks might only need a refresher or more targeted information about naloxone indications and usage.

In conjunction with targeted, patient-specific education, the LCS has the benefit of relationship building and prolonged face-to-face interaction with each participant. In a busy orthopedic trauma practice, this is critical. The LCS will meet with the participant each inpatient day and during three postoperative follow-up appointments. These follow-up appointments allow for a continuity of care from the LCS and increase the saliency of the administered opioid information. Participants are administered the same OLT at the 2-week and 12-week appointments and the LCS uses these survey results to adjust the postoperative follow-up opioid educational content. Participants are given review only where they need it.

The opioid information taught by the LCS includes information about proper disposal, common symptoms of opioid use, definition and use of naloxone, and signs of dependence and overdose. The LCS teaches behavioral wellness skills and opioid education orally and provides participants with a physical resource guide that includes visual representations and literature to take home.

### Referrals

Due to the nature of the LCS intervention (including time spent with participants, motivational interview training, and the survey instrumentation), the LCS often learns information about a participant that the rest of the healthcare team does not. When a participant identifies to the LCS a social or medical issue, the LCS can help arrange a referral for the participant. Some examples of the referrals that the LCS provided during a pilot study include mental health services, addiction medicine services, housing insecurity referrals, food insecurity referrals, and amputee support.

**Fig. 1:**
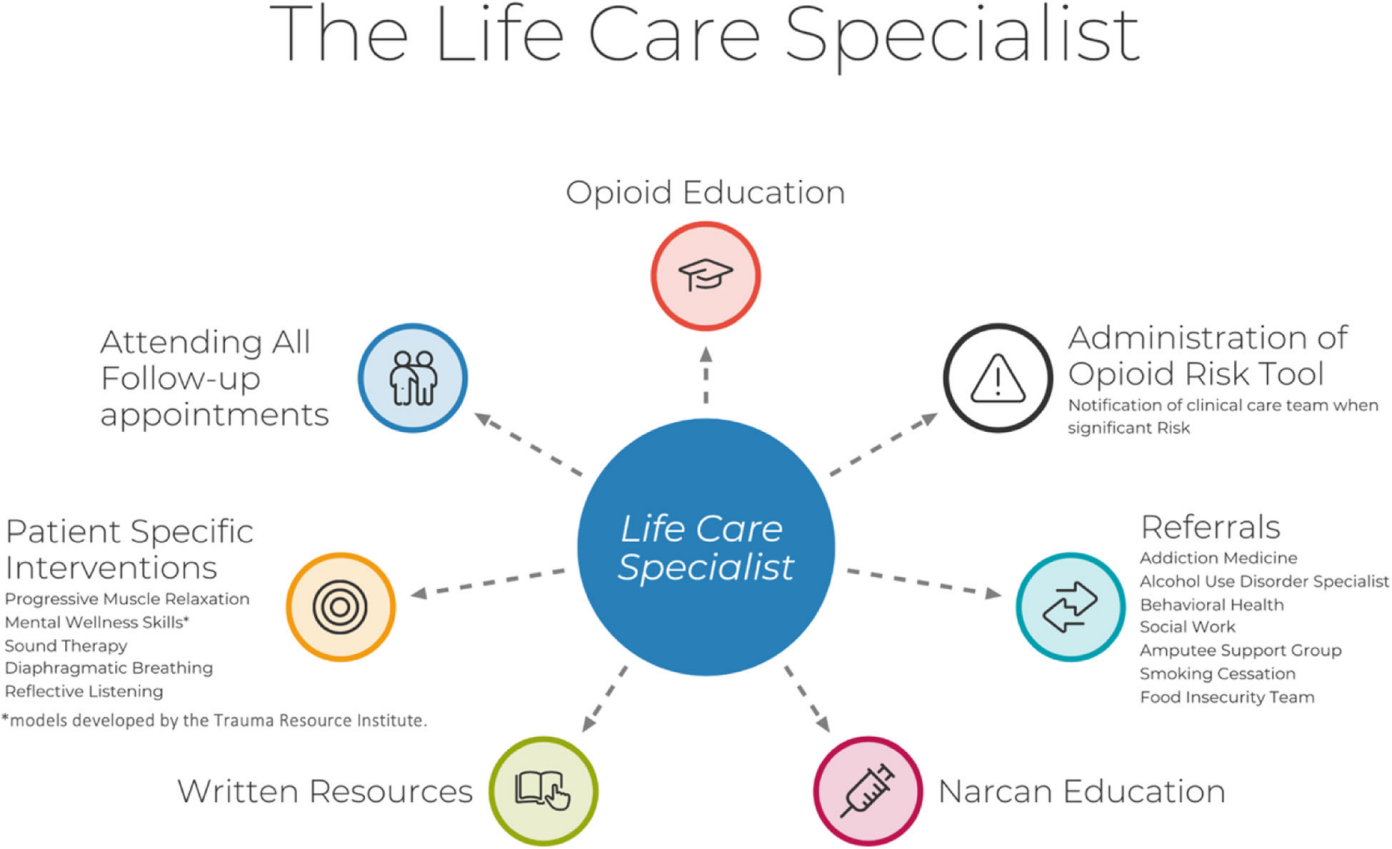
Life Care Specialist interventions

When giving referrals, the LCS works closely with physicians and nurses to make sure that the participant is a good fit for the referral program. All the LCS referrals at the study’s institution are pre-existing services offered by the hospital (Fig. 1).

Overall, as part of the daily LCS intervention, the participants will engage in behavioral pain management, opioid education, and harm-reduction strategies (naloxone education), while also being screened for eligibility for respective referrals for complex needs, such as mental health and substance use disorders. Upon discharge, each participant will be educated by the LCS on future available modes of contact (telephone, email, video-call, follow-up visits at 2, 6, and 12 weeks).

### Data collection

**Fig. 2:**
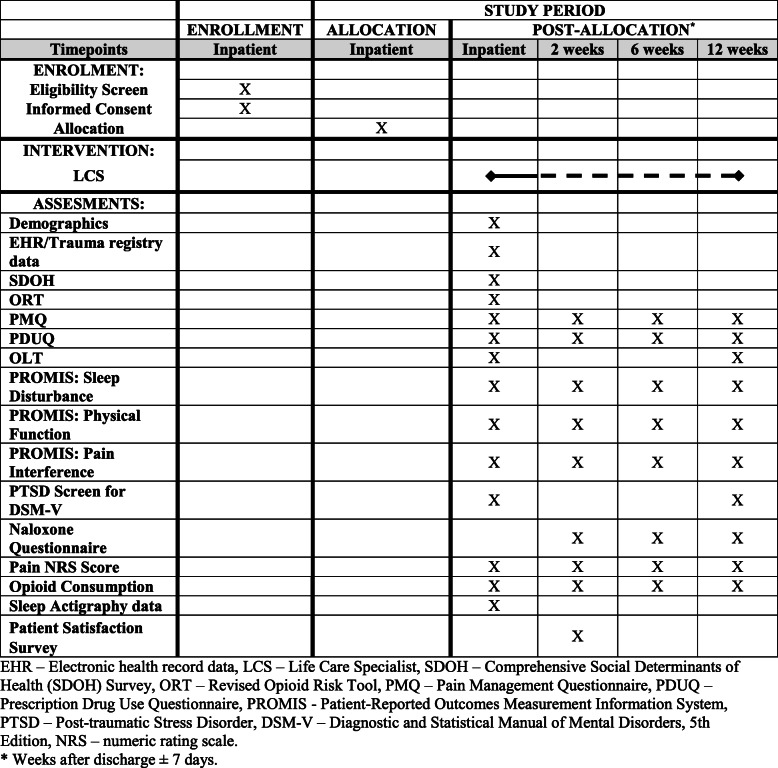
SPIRIT study schedule. EHR, Electronic health record data; LCS, Life Care Specialist; SDOH, Comprehensive Social Determinants of Health (SDOH) Survey; ORT, Revised Opioid Risk Tool; PMQ, Pain Management Questionnaire; PDUQ, Prescription Drug Use Questionnaire; PROMIS, Patient-Reported Outcomes Measurement Information System; PTSD, post-traumatic stress disorder; DSM-V, Diagnostic and Statistical Manual of Mental Disorders, 5th Edition; NRS, numeric rating scale. * Weeks after discharge ± 7 days

All data will be collected and stored via the Research Electronic Data Capture program (REDCap®) (Vanderbilt University, Nashville, TN, USA, 2020, version 9.1.15) and all participants will be subject to identical means and content of data collection by blinded research staff. Immediately after enrollment, research staff will administer survey instruments to all participants using tablets. Survey instruments include patient-reported outcomes (e.g., pain numeric rating scale (NRS), Patient-Reported Outcomes Measurement Information System (PROMIS)), screening assessments (e.g., Opioid Risk Tool (ORT), post-traumatic stress disorder screen (PTSD), Social Determinants of Health Survey (SDOH)), and participants’ understanding of opioid-related risks (e.g., Opioid Literacy Tool (OLT), Naloxone questionnaire). Demographic and clinical characteristics will be obtained from the Electronic Health Record (EHR) and institutional trauma registry. Opioid data will be captured using self-report measures and verified in both the EHR and PDMP. Objective outcomes related to pain and sleep will be captured using actigraphy devices that will be placed on participants’ non-injured wrists or ankles by study staff. All baseline measurements except the SDOH and ORT will be repeated on one, two, or all of the follow-up visits. Apart from the pain score and opioid utilization (pain score and opioid utilization during inpatient stay will be extracted from the EHR), all follow-up measurements will be collected directly from all enrolled participants by the research staff, during the 2, 6, and 12 weeks postoperative visit. The surveys will be administered at the follow-up intervals depicted in Fig. 2, which was designed in accordance with the Standard Protocol Items: Recommendations for Interventional Trials (SPIRIT) guidelines [42]. An example of all questionnaires and surveys can be reviewed in the Additional file 1. All participating staff have received formal training for all their tasks, and guides containing standard operating procedures are available on site on a secure intranet platform. If participants cannot physically travel to the clinic, appointments will be conducted over video conferencing and participants will have the option to complete surveys via email. To further minimize the rate of loss to follow-up, research staff will call participants to complete measures over the phone within 72 h of the scheduled appointment. In the event of no response, paper copies containing questionnaires and surveys will be mailed to the participants with prepaid postage for return.

### Measures

#### Pain numerical rating scale (NRS)

The NRS requires respondents to rate the intensity of their pain on a defined scale from 0, “no pain”, to 10, “the worst pain imaginable.” The NRS is a commonly used pain assessment tool in both clinical practice and research [43]. However, the NRS is a single static measure of pain and does not capture the biopsychosocial presentations of pain including physical functioning. As such a battery of objective (e.g., actigraphy) and patient-reported outcomes are needed to best ascertain patient participants’ pain experiences.

#### PROMIS Physical Function

PROMIS Physical Function measures participants’ self-reported capability to conduct physical activity. This includes capturing function in upper extremities and lower extremities (walking or mobility) as well as a respondent’s ability to conduct activities of daily living. There are 4-items on the short-form questionnaire and respondents report their capabilities to perform each task on a Likert scale from 5, “without any difficulty”, to 1, “unable to do.” All 4-items’ raw scores are summed before being transformed into *t*-scores ranging from 0 to 100. Higher scores are better and indicate greater physical function. The validated instrument is comparable to numerous legacy measures often used across diverse patient populations [44, 45].

#### PROMIS Pain Interference

The PROMIS Pain Interference scale assesses the extent to which pain impedes engagement with social, cognitive, emotional, physical, and recreational activities over the past 7 days. Pain interference is an essential aspect of pain management to capture in order to better understand how pain impacts the activities of individuals rather than subjective severity alone. On each of the scale’s 4-items respondents choose how much pain impeded a specific function or activity, ranging from 1, “not at all”, to 5, “very much.” Scores are summed across all items and transformed to a *t*-score ranging from 0 to 100, with lower *t*-scores indicating less interference due to pain. The PROMIS Pain Interference scale has been found to be comparable in responsiveness to traditional measures of pain interference used including the Brief Pain Inventory Interference subscale and the 36-Item Short Form Survey (SF-36) Bodily Pain scale [46–49].

#### PROMIS Sleep Disturbance

The PROMIS Sleep Disturbance examines respondent’s global severity of insomnia, sleep disruption, and sleep quality over the past 7 days. This PROMIS scale is more sensitive at detecting sleep problems than historical measures, such as the Pittsburgh Sleep Quality Index [50]. Again, each of the 4 Likert scale items’ raw score is converted to *t*-scores, ranging from 0 to 100. Like all PROMIS measures, *t*-scores are normed to the US population, with a mean of 50 and a standard deviation of 10 [51]. Lower scores indicate better sleep.

#### Opioid consumption

Opioid consumption will be examined both during hospitalization as well as throughout recovery up to 12 weeks. Opioid medication dosage will be transformed to a total universal measure known as morphine milligram equivalent (MME). Inpatient utilization will be extracted from the EHR by study staff upon participants’ discharge from the hospital. MME will be averaged over the length-of-stay (LOS) for a daily dosage, known as MME/day. Additionally, the study team will review participants’ EHR at each study time point up to 12 weeks to determine MME throughout postoperative recovery and rehabilitation.

### Relevant covariates

#### Comprehensive Social Determinants of Health Survey

This battery of measures assesses participants’ housing stability, financial health and comfort, education, community context, exposure to intimate partner violence, health literacy, family history of substance use, and adverse childhood experiences. These screening questions are routinely used by the team on other studies in the trauma service and align with recommendations from consensus groups [52, 53].

#### PTSD screen for DSM-V

This screener is a 5-item screening tool used to assess previous exposure to traumatic events and subsequent presence of the DSM-V diagnostic criteria for PTSD [54]. Each item respondents report “Yes” to can be scored as a point so that a minimum of 3 points is used in primary care settings to be considered probable PTSD.

#### Opioid Risk Tool (ORT)

The ORT is a self-reported measure used to ascertain a participant’s current and future risk of aberrant drug-related behaviors in patients prescribed opioid therapy. Recently a shortened revised ORT (the ORT-OUD) was developed [55, 56]. Across 9-items, this tool assesses family history of substance abuse, personal history of substance abuse, age range, and current psychological disease. Each endorsement is scored as 1 for a total score ranging from 0 to 9. Scores of 3 or greater are predictive of a high risk for opioid use disorder [48].

#### Electronic health record (EHR) data

Relevant health history will be derived from the electronic health record. Past and current diagnoses and medications pertaining to sleep (e.g., obesity, primary insomnia, narcolepsy, routine use of melatonin or other sleep aids), pain, and psychosocial conditions (e.g., major depressive disorder, antidepressants) will be abstracted. Additionally, all relevant comorbidities outlined in the Elixhauser Comorbidity Index will be screened for and collected from the electronic health record (e.g., COPD and AIDS). ASA physical status, injury severity score, smoking history, length of surgery, surgery type, and demographics (e.g., age, gender, body mass index, ethnicity) will also be abstracted from participants’ electronic health records by trained staff.

#### Naloxone questions

Each participant will complete a naloxone questionnaire at the end of the study period, evaluating knowledge and details of utilization. Naloxone procurement and understanding, captured in the EHR and measured using items from the validated Opioid Overdose Knowledge Scale and Opioid Overdose Attitudes Scale, respectively will also be assessed 2 weeks at follow-up [57].

#### Pain Management Questionnaire (PMQ) and Prescription Drug Use Questionnaire (PDUQ)

These questionnaires will collect longitudinal data on pain management behavior and prescription drug use throughout the study period The PMQ is a 26-item questionnaire where responses are given on a 5-point Likert scale where 0 = disagree and 4 = agree. Total scores range from 0 to 104, where higher scores indicate an increased risk of opioid misuse. The PDUQ is structured and administered in a similar fashion to assess the risk of drug misuse by collecting longitudinal data on prescription drug use during the study period.

#### Opioid Literacy Tool (OLT)

This survey is designed to assess the accuracy of knowledge about opioids (3 questions) and opioid-related risks (5 questions). Accuracy of opioid knowledge responses are given on a dichotomous scale (yes/no) and a pick-N scale, the latter of which will represent a percent accuracy of identified opioids and/or opioid-containing medication. Responses for accuracy of knowledge about opioid-related risks are given on a 7-point scale where 1 = definitely true and 7 = definitely false. For these 5 questions, a total score ranges from 5 to 35 and higher scores indicate improved literacy (accurate understanding of prescription opioid addiction-risk, opioid dependence, and risk of opioid overdose).

### Ancillary sleep actigraphy study

Wrist actigraphy is a valid and objective tool to measure activity patterns and sleep-related parameters, which has been used in patient populations with both acute and chronic pain [58, 59]. Actigraphy data includes objective quantitative measures of sleep, such as total sleep time, sleep latency, fragmentation, wake after sleep onset, and sleep efficiency. Additionally, activity level is also captured using wrist actigraphy, including total activity time, steps, physical activity intensity, and total energy expenditure.

In this study, actigraphy-based sleep and activity data (e.g., average hours of sleep, average daily steps) will be captured during inpatient hospitalization for all enrolled patients. After initial consent and enrollment, the research staff will provide participants with a screen-less wrist actigraphy device (GT3XP-BTLE, Actigraph, LLC, USA) to wear after surgery during their hospitalization. They will be trained by the research staff on how to wear it and the devices will be set to record in 30-s epochs at a medium sensitivity level for scoring sleep and wake time. Wear time validation will be accomplished using the Choi algorithm, as it more accurately estimates time worn accounting for forward and backward motions [60]. Participants will return their actigraphy devices at the 2-week follow-up appointment or be provided a pre-paid envelope to take home and mail back the device after their visit.

The sleep data will be computed using the Cole-Kripke algorithm, which accurately distinguishes sleep from wakefulness approximately 88% of the time [61]. Metabolic equivalent of tasks and energy expenditure will be measured with the Freedson algorithms [62]. By uniquely pairing actigraphy data with PROMIS patient-reported pain, sleep, and physical function measures, this study will be one of the first to provide a robust analysis of sleep quality and activity in tandem to pain presentations in adult trauma patient populations during hospitalization until discharge. These research efforts adhere to recommended best practices for using actigraphy to examine health outcomes [63].

### Outcomes

#### Primary outcomes

Patient-reported pain outcomes are the main outcomes of this study. Specifically, improvements in pain scores were captured on the NRS over the 12-week study period. Measuring the multidimensional nature of complex trauma pain is improved by using several assessments, each capturing unique theoretical domains and presentations of postoperative recovery. As such, improvements in PROMIS scores will be evaluated.

#### Secondary outcomes

The educational aspect of the intervention will be measured with the OLT and naloxone questionnaire at baseline and 12 weeks postoperatively and at each follow-up visit, respectively. Furthermore, the SDOH and ORT data will deliver insight into which patient profiles benefit most from the LCS intervention.

### Sample size

A sample of 200 provides 85% power (*α*=0.05) to detect a difference as small as 0.5 in the mean pain score between the intervention arm and the controls using repeated measures mixed-effects modeling (Power Analysis and Sample Size Software, NCSS, Kaysville, UT, USA). All analyses will be conducted in R (R Core Team, Vienna, Austria). The *glmnet* package will be used to determine which variables should be included in the regression models using a Least Absolute Shrinkage and Selection Operator (LASSO) approach, the *lme4* package will be employed to compute final regressions and assess fit [64, 65]. Post hoc power analyses will be conducted when exploring differences in outcomes based on demographics.

### Statistical analysis

A statistician blinded to the group allocation will perform the statistical analysis of all randomized patients.

To examine differences in patient-reported pain-related outcomes between those in the intervention compared to the control arm, a multivariable mixed-effects logistic regression model will be constructed to test the hypothesis that LCS intervention will yield lower average NRS pain scores and PROMIS scores postoperatively, compared control patients. A separate linear mixed-effects model will be constructed for each outcome. This modeling approach will enable the team to examine the mean differences in each patient-reported pain-related outcome while adjusting for covariates (e.g., mental health history, BMI, and surgery type). The mixed-effects modeling will account for potential collinearity between participants seen by the same surgeon by fitting a random effect for both factors. Models will be constructed using a LASSO method. The fit of the most appropriate and still parsimonious model produced by the LASSO will be assessed based on the Akaike Information Criterion (AIC) score.

For testing the hypothesis that participants in the intervention will report decreased utilization of opioid pain medications both in the inpatient and outpatient setting up to 12 weeks postoperatively, the same systematic machine learning guided model construction approach will be used. A separate mixed-effects model will be constructed to estimate differences in average MME/day during inpatient hospitalization as well as to estimate the difference in average MME/day over every study follow-up point up to 3 months postoperatively.

To explore the feasibility of utilizing actigraphy devices to capture postoperative functional outcomes among patients during their hospitalization and up to 2 weeks postoperatively following orthopedic trauma, generalized estimating equations (GEE) will be used. Activity will be measured as the average step per day and sleep will be measured as average nightly sleep efficiency and duration. Separate GEE will be computed for each actigraphy-related measure. In the event of a significant change in any of the objective outcomes, a linear mixed-effect model will be constructed to ascertain any significant differences in actigraphy collected outcomes exist between participants in the intervention and the control arms over the two-week postoperative period. The linear mixed-effects models will also include a random effect to account for within-participant collinearity and be able to adjust for changes covarying daily pain scores collected via SMS.

### Interim analysis

An interim analysis of the collected data will allow for quality assurance of collected measures and outcomes, in addition to dual-data entry by research staff. This is scheduled during the half-way point of enrollment (50 participants for each group) and only the principal investigator and the blinded statistician will have access to these results.

### Data monitoring

Due to the known minimal risk of this study, a data monitoring committee is not warranted per our institution’s IRB.

### Recruitment feasibility

As a level I trauma center the recruitment facility is not only one of the largest volume trauma centers in the region but also is the fifth-largest public hospital in the United States. The orthopedic trauma service conducts over 5600 surgical procedures annually. Collectively, the volume of procedures, the diversity of patients seen at these the health system, and the investigators on this study who are active clinicians serving this patient population indicates the feasibility of recruiting a representative number of participants to meet recruitment goals.

### Dissemination

A multipronged dissemination approach that incorporates traditional academic peer-reviewed outlets, such as conferences and journal articles will be utilized. We will produce at least 2 peer-reviewed data-based manuscripts. Additionally, we will meet with clinicians to review findings and contextualize results.

## Discussion

More than 125 people in the USA die each day from an opioid-involved overdose [2]. Despite healthcare interventions, initiatives, and prescriber-targeted regulations the staggering death toll from opioid-involved overdoses continues to rise without abatement [66–69]. Given the incongruous trends of a relative decrease in prescription rate [70] versus an increasing opioid-related fatality rate [3, 71], we acknowledge the apparent importance of patient-centered interventions to obviate prescription opioid-related morbidity [72]. In this prospective, blinded, randomized controlled trial, we propose the addition of a novel member of the healthcare team, an LCS, to direct that patient-centric approach in the post-orthopedic trauma patient.

Several studies have demonstrated that unidimensional solutions to multifaceted public health issues often yield undesired results [18, 73]. Uniquely, the LCS intervention incorporates patient-centric opioid education, with guided approaches to alternative methods of pain management in tandem to prescription medication. The LCS teaches progressive muscle relaxation, resiliency, and encounters are guided through structured motivational interviews and reflexive listening. The relationship that the LCS builds with the patient can uncover underlying risk factors for opioid misuse and abuse and can safely direct patients towards various substance use- and abuse related support and cessation programs, which have shown firm evidence of co-dependent efficacy in the treatment of opioid use disorder [74, 75].

Over the last year our interdisciplinary team has worked with community partners at the Christopher Wolf Crusade to train and implement four Life Care Specialists into practice at a level I trauma center in Atlanta. Pilot results indicate that out of the 122 participants seen by Life Care Specialists after being admitted due to an orthopedic trauma, 48% screened positive for being at moderate to high risk for opioid misuse based on the validated Opioid Risk Tool. Two thirds of participants in the pilot identified as being Hispanic, Black, or African American. A quarter (25%) of participants reported being unstably housed. Over half screened positive on Hunger Vital Signs (51.6%) for food insecurity. On average, patients reported utilizing approximately 2 pain management interventions the Life Care Specialists trained them on, with the most frequently used approaches being progressive muscle relaxation (37.7%) and music for distraction (31.1%). Narcan training and prescriptions were provided to all participants. At time of discharge, nearly all participants (99%) agreed that working with a Life Care Specialists was helpful in managing their pain. These results suggest that when interpersonal interventions incorporate the educational and cultural context of pain, patients benefit. After orthopedic trauma, it is possible to provide pain management and opioid education that improves post-trauma pain experience and mitigates opioid-related risks through a patient-centered approach. These promising preliminary findings not only demonstrated the feasibility and acceptability of integrating a Life Care Specialist into clinical practice but also informed the present randomized controlled trial study design.

In summary, this study will assess the feasibility and efficacy of the LCS intervention in a longitudinal orthopedic trauma setting. Given the increasing necessity for personalized non-opioid pain management strategies, the findings from this study will provide imperative scientific and clinical evidence on the efficacy and impact of an individualized, multimodal, non-pharmacologic, behavioral-based pain management intervention, to achieve opioid-related risk-mitigation in a high-risk population. Moreover, the final aim will provide early evidence into which patients benefit most from LCS intervention. Finally, the findings of this study, coupled with the readily accessible conceptual framework of this academic-community partnership may facilitate the expansion of the LCS program, and thereby continuously foster health equity by translating research into practice and policy.

## Trial status

This study was approved by the Institutional Review Board of Emory University (IRB00115061) on 6/9/2021 (latest version) and registered at ClinicalTrials.gov (NCT04154384) on 11/6/2019 (last updated on 6/10/2021).

The recruitment of participants started on May 28th, 2021. The anticipated recruitment period is 3 months. This protocol is version 1.

## Supplementary Information

**Additional file 1.** Informed consent

## Data Availability

The datasets used and/or analyzed during the current study are available from the corresponding author on reasonable request.

## Abbreviations

CWC: Christopher Wolf Crusade
LCS: Life Care Specialist
EHR: Electronic health record data
RCT: Randomized controlled trial
SPIRIT: Standard Protocol Items: Recommendations for Interventional Trials
CRC: Clinical research coordinator
REDCap®: Research Electronic Data Capture Program
CRM: Community Resiliency Model
ORT: Revised Opioid Risk Tool
PMR: Progressive muscle relaxation
SDOH: Social Determinants of Health
OLT: Opioid Literacy Tool
PDMP: Prescription Drug Monitoring Program
PMQ: Pain Management Questionnaire
PDUQ: Prescription Drug Use Questionnaire
PTSD: Post-traumatic stress disorder
DSM-V: Diagnostic and Statistical Manual of Mental Disorders, 5th Edition
PROMIS: Patient-Reported Outcomes Measurement Information System
BMI: Body mass index
LOS: Length of stay
NRS: Numeric rating scale
MME: Morphine milligram equivalent
LASSO: Least Absolute Shrinkage and Selection Operator
GEE: Generalized estimating equations
AIC: Akaike Information Criterion
