# Supplementary material for: The effect of a Life Care Specialist on pain management and opioid-related outcomes among patients with orthopedic trauma: study protocol for a randomized controlled trial
Source: Trials. 2021 Nov 27;22:858. doi: 10.1186/s13063-021-05841-1 (PMC8626911; doi:10.1186/s13063-021-05841-1)
Supplement: Supplementary file 1 — Additional file 1. Informed consent [file 13063_2021_5841_MOESM1_ESM.pdf]

Informed Consent

Please complete the survey below.

Thank you!

Consent

MOD0010-IRB00115061
IRB Approved
4/11/2021

You Are Being Asked to Be in a Research Study

Concise presentation of key concepts

You are being asked to be in a research study. A research study is designed to answer a scientific question. If you agree to be in the study you will be one of 500 people who are being studied, at Emory.

Why is this study being done?

This study is being done to learn more about Life Care Specialist (LCS) position and the value it provides to the patient and the healthcare setting in reducing misuse of prescriptions. Additionally, the resources required to carry it through. You are being asked to be in this research study because you have had or will be having surgery with one of our Orthopaedic surgeons.

Do you have to be in the study?

It is your decision to be part of this research study. You do not have to be in it. Your choice will not affect your access to medical care for your condition. Before you make your decision, you should take time to learn about the study.

What do I have to do if I choose to participate in this study?

If you are eligible and want to be a part of the study, you have an equal chance of being assigned to any one of the two groups.

If you are in the control group you will complete 4 study visits.

If you are in the treatment group you will complete 7 study visits.

The researchers will ask you to answer questionnaires and participate in the management for your pain.

How is this study going to help you?

If you are in the study, you will be helping the researchers answer the study question. To uncover the strengths and weaknesses of the Life Care Specialist position. In addition, the value it provides to the patient and healthcare setting and the resources required to carry through.

Alternatives to Joining This Study

If you decide not to enter this study, there is care available to you outside of this research study. You do not have to be in this study to be treated for your injury.

Costs

There are no costs for your participation on this study.

What Should I Do Next?

Read this form, or have it read to you. Make sure the study doctor or study staff explains the study to you. Ask questions (e.g., about exact time commitment, about unfamiliar words, more details on specific procedures, etc.) Take time to consider this, and talk about it with your family and friends.

Emory University and Grady Health System
Consent to be a Research Subject / HIPAA Authorization

Title: Life Care Specialist (LCS)

Principal Investigator: Dr. Mara Schenker

Sponsor: Christopher Wolf Crusade (CWC)

Investigator-Sponsor: Cammie Wolf Rice

Introduction
You are being asked to be in a medical research study. This form is designed to tell you everything you need to think about before you decide if you want to be a part of the study. **It is entirely your choice. If you decide to take part, you can change your mind later on and withdraw from the research study.** The decision to join or not join the research study will not cause you to lose any medical benefits. If you decide not to take part in this study, your doctor will continue to treat you.

Before making your decision:

- Please carefully read this form or have it read to you
- Please listen to the study doctor or study staff explain the study to you
- Please ask questions about anything that is not clear

You can take a copy of this consent form, to keep. Feel free to take your time thinking about whether you would like to participate. You may wish to discuss your decision with family or friends. Do not sign this consent form unless you have had a chance to ask questions and get answers that make sense to you. By signing this form you will not give up any legal rights.

A description of this clinical trial will be available on http://www.ClinicalTrials.gov, as required by U.S. law. This Web site will not include information that can identify you. At most the Web site will include a summary of the results. You can search this Web site at any time.

What is the purpose of this study?
The main focus of the Christopher Wolf Crusade (CWC) is prevention through the use of a Life Care Specialist (LCS). We are working with key stakeholders in the opioid epidemic to develop an official pain management protocol, as well as conducting an introductory study for a new field of Pain Management.

The goal is to see Life Care Specialists (LCS) staffed in hospitals to focus on pain management and addiction prevention for patients. The LCS position does not currently exist in the healthcare field. Additionally, it will help provide information about the resources that are needed to carry it through.

What will I be asked to do?
If you agree to be a part of the study and are randomized to the control group you will have 4 study visits: hospital admission, 2 week, 6 week and 3 month follow-up.

If you agree to be a part of the study and are randomized to the treatment group you will have 7 study visits: hospital admission, 2 week, 6 week and 3 month follow-up. You'll also complete LCS interventions at your 2 week, 6 week and 3 month follow-ups.

You will be asked to complete questionnaires in person and via text messaging (SMS) that will give us information about your general health and your pain at each appointment with your surgeon. If any questions make you too uncomfortable to answer, you can skip them. Additionally, we will collect information from your hospital admission.

You will be asked to complete the following questionnaires that ask questions about your pain and your health. The questionnaires are:

- Demographics
- Comprehensive social determinants of health survey (SDOH)
- Opioid Risk Tool (ORT)
- Pain Management Questionnaire (PMQ)
- Prescription Drug Use Questionnaire (PDUQ)
- PROMIS: Sleep Disturbance
- PROMIS: Physical Function
- PROMIS: Pain Interference
- Health Misinformation
- Pain Management Survey
- Patient Satisfaction Survey
- Narcan Questionnaire
- PTSD Screener

The following will be collected via text (SMS):

- Actigraphy collects sleep and activity data
- NRS Pain Scores
- Opioid Use

<u>Who owns my study information?</u>
If you join this study, you will be donating your study information. If you withdraw from the study, data that was already collected may be still be used for this study. Study reporting will not identify any one person.

<u>What are the possible risks and discomforts?</u>
There may be side effects from the study or procedures that are not known at this time. Rare but possible risks include: Breach of confidentiality; however, we will be sure to keep all of your protected health information in a password protected, encrypted database only accessible to study team members.

We are asking you to complete questionnaires asking about many social factors that may be sensitive and potentially emotionally upsetting. This includes information about your socioeconomic position, race, ethnic group, cultural context, gender, sexual orientation, alcohol and drug abuse, family and domestic abuse, social relationships, and residential and community context. The questionnaires will be completed in-person, over the phone and/or text messaging (SMS). If any questions make you feel uncomfortable, you can skip them.

Though you will be using pain medications as prescribed, the doctors acknowledge that you are asked to quantify the consumption of a controlled substance, which may be a sensitive matter. Thus, in order to further protect your confidentiality of responses to survey questions, the text messaging (SMS) and online survey responses will be stored as a set of numbers only, without identifying the question to which those numbers pertain. This data will be stored in an encrypted fashion on a commercial cloud server, which is password protected and only accessible by Emory research staff. The survey key linking the questions to the your responses will also only be available to Emory investigators. All protected health information for this study, with the exception of a cell phone number, as it is required to send you text

message survey questions, will be stored separately from the daily survey responses. Your participation in the text message survey may be stopped at any time by responding 'Stop' to a survey question.

Will I benefit directly from the study?
The goal is to introduce a Life Care Specialist (LCS) as an integral member of the clinical team, with a focus on "pain coaching" for trauma patients. The study results may be used to help others in the future.

Will I be compensated for my time and effort?
On your first visit, you will get a gift bag (blanket, bag, water bottle, pen, T-shirt, hat, toothbrush and toothpaste).

At your 2 week and 6 week visit, you will get a vendor-issued gift card, with a cash value of less than $10.

At the conclusion of the study (3 months), you will get a Visa gift card, with a value of $25.

How will you protect my private information that you collect in this study?
Whenever possible, a study number, rather than your name, will be used on study records. Your name and other identifying information will not appear when we present or publish the study results.

Storing and Sharing your Information
De-identified data from this study (data that has been stripped of all information that can identify you), may be placed into public databases where, in addition to having no direct identifiers, researchers will need to sign data use agreements before accessing the data. We will remove or code any personal information that could identify you before your information is shared. This will ensure that, by current scientific standards and known methods, it is extremely unlikely that anyone would be able to identify you from the information we share. Despite these measures, we cannot guarantee anonymity of your personal data.

Your data from this study may be useful for other research being done by investigators at Emory or elsewhere. To help further science, we may provide your de-identified data to other researchers. If we do, we will not include any information that could identify you. If your data is labeled with your study ID, we will not allow the other investigators to link that ID to your identifiable information.

In general, we will not give you any individual results from the study of the data you give us. If we find something of urgent medical importance to you, we will inform you, although we expect that this will be a very rare occurrence.

Medical Record
If you have been an Emory and Grady Health System patient before, then you already have an Emory and Grady Health System medical record. If you have never been an Emory and Grady Health System patient, you do not have one. An Emory and Grady Health System medical record will be made for you if an Emory and Grady Health System provider or facility gives you any services or procedures for this study.

Copies of the consent form/HIPAA authorization that you sign will be put in any Emory and Grady Health System medical record you have now or any time during the study.

Tests and procedures done at non-Emory and Grady Health System places may not become part of your Emory and Grady Health System medical record. Also, if you decide to be in this study, it is up to you to let your other health providers know.

Costs
There will be no costs to you for participating in this study, other than basic expenses like transportation. You will not be charged for any of the research activities.

Withdrawal from the Study
You have the right to leave a study at any time without penalty.

The researchers also have the right to stop your participation in this study without your consent for any reason, especially if they believe it is in your best interest or if you were to object to any future changes that may be made in the study plan.

Authorization to Use and Disclose Protected Health Information

The privacy of your health information is important to us. We call your health information that identifies you, your "protected health information" or "PHI." To protect your PHI, we will follow federal and state privacy laws, including the Health Insurance Portability and Accountability Act and regulations (HIPAA). We refer to all of these laws as the "Privacy Rules." Here we let you know how we will use and disclose your PHI for the study.

PHI that Will be Used/Disclosed:
The PHI that we will use or share for the main research study includes:

- Medical information about you including your medical history and present/past medications.
- Results of exams, procedures and tests you have before and during the study.
- Information related to your mental health.
- Information related to your pain management.

Purposes for Which Your PHI Will be Used/Disclosed:
We will use and share your PHI for the conduct and oversight of the research study. We will use and share your PHI to provide you with study related treatment and for payment for such treatment. We will also use and share your PHI to conduct normal business operations. We may share your PHI with other people and places that help us conduct or carry out the study, such as laboratories, data management centers, data monitors, contract research organizations, Institutional Review Boards (IRBs) and other study sites. If you leave the study, we may use your PHI to determine your health, vital status or contact information.

Use and Disclosure of Your Information That is Required by Law:
We will use and disclose your PHI when we are required to do so by law. This includes laws that require us to report child abuse or abuse of elderly or disabled adults. We will also comply with legal requests or orders that require us to disclose your PHI. These include subpoenas or court orders.

Authorization to Use PHI is Required to Participate:
By signing this form, you give us permission to use and share your PHI as described in this document. You do not have to sign this form. If you do not sign this form, you may still receive non-research related treatment.

People Who will Use/Disclose Your PHI:
The following people and groups will use and disclose your PHI in connection with the research study:

- The Principal Investigator and the research staff will use and disclose your PHI to conduct the study and give you study related treatment.
- The Principal Investigator and research staff will share your PHI with other people and groups to help conduct the study or to provide oversight for the study.

- Christopher Wolf Crusade (CWC) is the Sponsor of the study. The Sponsor may use and disclose your PHI to make sure the research is done correctly and to collect and analyze the results of the research. The Sponsor may disclose your PHI to other people and groups like study monitors to help conduct the study or to provide oversight for the study.
- The following people and groups will use your PHI to make sure the research is done correctly and safely:
    - Emory and Grady Health System offices that are part of the Human Research Participant Protection Program and those that are involved in study administration and billing. These include the Emory IRB, the Grady Research Oversight Committee, the Emory Research and Healthcare Compliance Offices, and the Emory Office for Clinical Research.
    - Public health agencies.
    - Research monitors and reviewer.
    - Accreditation agencies.

Expiration of Your Authorization
Your PHI will be used until this research study ends.

Revoking Your Authorization
If you sign this form, at any time later you may revoke (take back) your permission to use your information. If you want to do this, you must contact Erika Ortega at 404-251-8953.

At that point, the researchers would not collect any more of your PHI. But they may use or disclose the information you already gave them so they can follow the law, protect your safety, or make sure that the study was done properly and the data is correct. If you revoke your authorization you will not be able to stay in the study.

Other Items You Should Know about Your Privacy
Not all people and entities are covered by the Privacy Rules. HIPAA only applies to health care providers, health care payers, and health care clearinghouses. If we disclose your information to people who are not covered by the Privacy Rules, including HIPAA, then your information won't be protected by the Privacy Rules. People who do not have to follow the Privacy rules can use or disclose your information with others without your permission if they are allowed to do so by the laws that cover them. The Sponsor, and people and companies working with the Sponsor on this study are not covered by the Privacy Rules. They will only use and disclose your information as described in this Consent and Authorization.

To maintain the integrity of this research study, you generally will not have access to your PHI related to this research until the study is complete. When the study ends, and at your request, you generally will have access to your PHI that we maintain in a designated record set. A designated record set is data that includes medical information or billing records that your health care providers use to make decisions about you. If it is necessary for your health care, your health information will be provided to your doctor.

We may remove identifying information from your PHI. Once we do this, the remaining information will not be subject to the Privacy Rules. Information without identifiers may be used or disclosed with other people or organizations for purposes besides this study.

<u>Contact Information</u>
Contact Erika Ortega at 404-251-8953:

- if you have any questions about this study or your part in it,
- if you have questions, concerns or complaints about the research

Contact the Emory University Institutional Review Board at 404-712-0720 or 877-503-9797 or irb@emory.edu:

- if you have questions about your rights as a research participant.
- if you have questions, concerns or complaints about the research.
- You may also let the IRB know about your experience as a research participant through our Research Participant Survey at http://www.surveymonkey.com/s/6ZDMW75.

If you are a patient receiving care from the Grady Health System and have a question about your rights, you may contact the Office of Research Administration at research@gmh.edu.

Consent and Authorization

TO BE FILLED OUT BY SUBJECT ONLY

Please **print** your name, **sign**, and **date** below if you agree to be in this research study. By signing this consent and authorization form, you will not give up any of your legal rights. We will give you a copy of the signed form to keep.

____
Patient Name

____ ______________
Patient Signature (18 or older and able to consent) **Date** **Time**

TO BE FILLED OUT BY STUDY TEAM ONLY

____
Name of Person Conducting Informed Consent Discussion

____ ______________
Signature of Person Conducting Informed Consent Discussion **Date** **Time**

1) First Name of Patient

____

2) Last Name of Patient

____

3) Patient Signature

____

4) Today's Date

____

5) Name of Person Conducting Informed Consent Discussion

____

6) Signature of Person Conducting Informed Consent Discussion

____

7) Today's Date

____

Participant Contact Information

Please complete the survey below.

Thank you!

1) First Name ________________________

2) Last Name ________________________

3) Your email address ________________________

4) Your cell phone number ________________________

5) Your landline phone number ________________________

6) Your mailing address ________________________

7) Emergency contact name ________________________

8) Emergency contact phone number ________________________

9) Emergency contact email: ________________________

10) Hospital Account Number ________________________

11) MRN ________________________

Opioid Risk Tool

Please complete the survey below.

Thank you!

1) Has anyone in your family (parents or siblings) ever had problems with alcohol use?
◯ Yes
◯ No

2) Has anyone in your family (parents or siblings) ever had problems with illegal drug use?
◯ Yes
◯ No

3) Has anyone in your family (parents or siblings) ever had problems with prescription drug use?
◯ Yes
◯ No

4) Have you ever had problems in the past with alcohol use?
◯ Yes
◯ No

5) Have you ever used or had problems using illegal drugs?
◯ Yes
◯ No

6) Have you ever had problems in the past with prescription medication?
◯ Yes
◯ No

7) Are you between 16 - 45 years old?
◯ Yes
◯ No

8) Have you ever been diagnosed with one or more of the following:

attention deficit disorder obsessive compulsive disorder bipolar disorder schizophrenia
◯ Yes
◯ No

9) Have you ever been diagnosed with depression?
◯ Yes
◯ No

10) Revised ORT score:

____
((≤2 low risk; ≥3 = high risk))

SDOH survey

Please complete the survey below.

Thank you!

Which of the following best describes people CURRENTLY living with you: (check all that apply)

- ☐ alone
- ☐ significant other
- ☐ spouse
- ☐ children
- ☐ family members
- ☐ friend
- ☐ parent
- ☐ roommate or housemate

Which of the following best describes where you CURRENTLY live: (select one)

- ◯ apartment (rent)
- ◯ private residence
- ◯ assisted living
- ◯ group home
- ◯ homeless
- ◯ hotel
- ◯ nursing home
- ◯ other group related setting
- ◯ shelter
- ◯ temporary

Was there ever a time you didn't have your own place to stay, were homeless, or stayed in a shelter?

- ◯ Yes
- ◯ No

Social Determinants of Health

Has lack of transportation (not having a car, a ride, or access to public transportation) kept you from getting to:

	Yes	No
Medical appointments or pharmacies to fill prescriptions?	◯	◯

Has your phone number changed within the last 12 months?

- ◯ Yes
- ◯ No

What is the highest level of school that you finished?

- ◯ High school diploma or equivalency (GED)
- ◯ Associate degree (junior college)
- ◯ Bachelor's degree
- ◯ Master's degree
- ◯ Doctorate
- ◯ Professional (MD, JD, DDS, etc..)
- ◯ Other
- ◯ None of the above (less than high school)

Question	Options
Which of the following best describes your main daily activities and/ or responsibilities BEFORE YOUR TRAUMA?	◯ Working full time<br>◯ Working part-time<br>◯ Working part-time multiple jobs<br>◯ Unemployed or laid off<br>◯ Looking for work<br>◯ Keeping house or raising children full time<br>◯ Retired<br>◯ Disability<br>◯ Student<br>◯ Taking time off, still employed<br>◯ Light duty or part time work, still employed<br>◯ Medical leave, still employed<br>◯ Other
Have you ever been convincted of a crime? (misdemeanor or felony)?	◯ Yes<br>◯ No
How hard is it for you to pay for the very basics, like food, housing, medical care, and heating?	◯ Very hard<br>◯ Somewhat hard<br>◯ Not hard at all
In the past 12 months, was there anytime when you could not get a prescription medicine because you could not afford it?	◯ Yes<br>◯ No
In the past 12 months, has the food you bought not lasted and you didn't have money to get more?	◯ Never true<br>◯ Sometimes true<br>◯ Often true<br>◯ Patient refused<br>◯ Not asked
In the past 12 months, have you worried about whether food would run out before you got money to buy more?	◯ Yes<br>◯ No

COMMUNITY: Do you feel safe in the following places...

	Yes	No
In your home?	◯	◯
In your neighborhood?	◯	◯
To walk and exercise in your neighborhood?	◯	◯

Question	Options
Do you ever feel judged in your neighborhood based on your culture, race, religion, or ethnicity?	◯ Yes<br>◯ No
Do you consider yourself to be:	◯ Heterosexual (straight)<br>◯ Homosexual<br>◯ Bisexual<br>◯ Prefer not to answer

INTIMATE PARTNER VIOLENCE: The following questions are sensitive, and address violence that you may have experienced in your home or with your partner. Within the PAST YEAR,

	Yes	No	Patient Refused	Not Asked
Have you been humiliated or emotionally abused in other ways by your partner or ex-partner?	◯	◯	◯	◯
Have you been afraid of your partner or ex-partner?	◯	◯	◯	◯
Have you been raped or forced to have any kind of sexual activity by your partner or ex-partner?	◯	◯	◯	◯
Have you been kicked, hit, slapped, or otherwise physically hurt by your partner or ex-partner?	◯	◯	◯	◯

Do you have a primary care physician?

◯ Yes
◯ No

Health Literacy

	All of the time	Most of the time	Some of the time	A little of the time	None of the time
How often do you have someone help you read hospital materials?	◯	◯	◯	◯	◯
How often do you have problems learning about medical conditions because of difficulty understanding written information?	◯	◯	◯	◯	◯
How confident are you filling out forms by yourself?	◯	◯	◯	◯	◯

Have you ever had a substance abuse problem?

◯ Yes
◯ No

Have you ever been treated for your substance abuse problem?

◯ Yes
◯ No

Does anyone in your family have a problem with drugs or alcohol?

◯ Yes
◯ No

Does your spouse or partner have problems with drugs or alcohol?

◯ Yes
◯ No
◯ N/A

Have you ever, or do you currently smoke cigarettes or vape?	◯ Current, Every day<br>◯ Current, Some days<br>◯ Current, Unknown how often<br>◯ Former<br>◯ Never
Have you ever had an alcoholic drink?	◯ Yes<br>◯ No
How often do you have a drink containing alcohol?	◯ Monthly or less<br>◯ 2-4 times/month<br>◯ 2-3 times/week<br>◯ 4 or more times/week<br>◯ I formerly drank alcohol, but currently do not
How many standard drinks containing alcohol do you have on a typical day when you are drinking?	◯ 0 - 2<br>◯ 3 or 4<br>◯ 5 or 6<br>◯ 7 to 9<br>◯ 10 or more
How often do you have six or more drinks containing alcohol on one occasion?	◯ Never<br>◯ Less than Monthly<br>◯ Monthly<br>◯ Weekly<br>◯ Daily<br>◯ Almost Daily
Have you ever used pain medication (opioid or narcotic) prior to this trauma?	◯ Yes<br>◯ No
How did you get the pain medications (check all that apply):	☐ prescribed by a doctor<br>☐ borrowed from family members or friends<br>☐ bought on the street (other than in the pharmacy)
Did your doctor tell you when and how to stop taking the medication?	◯ Yes<br>◯ No
Did your doctor also prescribe naloxone (Narcan) or tell you how to get this medication?	◯ Yes<br>◯ No
Has a physician ever told you you were dependent on pain mediciation (opioid, narcotic)?	◯ Yes<br>◯ No
In the last 6 months have you ever visited an emergency department because of pain?	◯ Yes<br>◯ No

Have you used any of these EVER in your life? (check all that apply)

- ☐ None
- ☐ Marijuana (cannabis, THC oil, dabbing)
- ☐ Synthetic marijuana (spice)
- ☐ Cocaine
- ☐ Heroin
- ☐ Hallucinogens (LSD, PCP, peyote, mescaline, psilocybin mushrooms, and Ecstasy)
- ☐ Inhalants (nitrous oxide, amyl nitrate, cleaning fluids, gasoline, spray paints, aerosol sprays, glue))
- ☐ Stimulants (meth, amphetamines, etc)

Have you used any of these in the past MONTH? (check all that apply)

- ☐ None
- ☐ Marijuana (cannabis, THC oil, dabbing)
- ☐ Synthetic marijuana (spice)
- ☐ Cocaine
- ☐ Heroin
- ☐ Hallucinogens (LSD, PCP, peyote, mescaline, psilocybin mushrooms, and Ecstasy)
- ☐ Inhalants (nitrous oxide, amyl nitrate, cleaning fluids, gasoline, spray paints, aerosol sprays, glue))
- ☐ Stimulants (meth, amphetamines, etc)

How old were you when you first used heroin?

____

How many years have you been using heroin?

____

During the heaviest period of use, how frequently were you using heroin?

- ○ Multiple times a day
- ○ Every day
- ○ Several times a week
- ○ Once a week
- ○ A few times a month
- ○ Less than a few times a month

Have you experienced withdrawal symptoms (anxiety, fatigue, sweating, vomiting, depression, seizures, and hallucinations) from heroin, narcotics, or opioids?

- ○ Yes
- ○ No

In the last month, how often have you felt?

	Not at all	Several days	More than half the days	Nearly every day
Little interest or pleasure doing things	○	○	○	○
Feeling down, depressed, or hopeless	○	○	○	○

Do you feel stress -- tense, nervous, anxious or unable to sleep at night -- because your mind is troubled?

- ○ Not at all
- ○ Only a little
- ○ To some extent
- ○ Rather much
- ○ Very much

SUICIDAL IDEATION and ADVERSE CHILDHOOD EXPERIENCES: The following questions may be very sensitive to a past experience. If you would like immediate referral to a specialist regarding these feelings, please let us know right away:

	Yes	No
Have you ever made a suicide attempt?	◯	◯
Have you ever made a suicide attempt in the past 12 months?	◯	◯
Have you had thoughts about killing yourself in the past 12 months?	◯	◯
When you were growing up in the first 18 years of your life, did you often feel like no one in your family loved you, thought you were important or special, or your family did not look out for each other, feel close to each other, or support each other?	◯	◯
When you were growing up in the first 18 years of your life, did an adult or person at least 5 years older than you ever....touch or fondle you or have you touch their body in a sexual way? Or try to actually have oral, anal or vaginal sex with you?	◯	◯

Have you ever been diagnosed with the following neurologic, muscle or mental illnesses? (Please select all that apply)

- ☐ Neurodevelopment disorders (intellectual disability, global development delay, speech disorder, autism, attention deficit disorder)
- ☐ Schizophrenia
- ☐ Bipolar and related disorder
- ☐ Anxiety disorders (generalized anxiety disorder, agoraphobia, social anxiety, phobias, panic attacks, separation anxiety disorder)
- ☐ Trauma and stressor related disorder (acute stress disorder, adjustment disorder, PTSD)
- ☐ Dissociative disorders (dissociative amnesia, depersonalization disorder)
- ☐ Eating disorder
- ☐ Personality disorder (antisocial, avoidant, borderline, dependent, histrionic, narcissistic, obsessive-compulsive disorder, paranoid, schizoid, schizotypal)
- ☐ Depression, MDD, Adjustment Disorder, Cyclothymic Disorder

Opioid Literacy Tool

Please complete the survey below.

Thank you!

---

1) Do you know what an opioid is?
- ◯ Yes
- ◯ No

---

2) Do you think you are taking an opioid?
- ◯ Yes
- ◯ No
- ◯ Not sure/I don't know

---

3) Which of the following are opioids?
- ☐ Tylenol (Acetaminophen)
- ☐ Narcan (Naloxone)
- ☐ Motrin (Ibuprofen)
- ☐ Aleve (Naproxen)
- ☐ Naprosyn (Naproxen)
- ☐ Toradol (Ketorolac)
- ☐ Ultram (Tramadol)
- ☐ Marijuana (Cannabis)
- ☐ Cocaine
- ☐ Heroin
- ☐ Percocet (Oxycodone/Acetaminophen)
- ☐ OxyContin (Oxycodone)
- ☐ MS Contin (Morphine)
- ☐ Duramorph (Morphine)
- ☐ Dilaudid (Hydromorphone)
- ☐ Duragesic (Fentanyl)
- ☐ Lortab (Hydrocodone/Acetaminophen)
- ☐ Tylenol #3 (Codeine/Acetaminophen)
- ☐ Voltaren (Diclofenac)

---

4) When I take more opioid pain medicine, my risk of getting addicted does not change.
- ◯ 1 Definitely True
- ◯ 2
- ◯ 3
- ◯ 4
- ◯ 5
- ◯ 6
- ◯ 7 Definitely False

---

5) People with severe pain that need opioids have trouble getting prescriptions because the drugs are all used up by addicts.
- ◯ 1 Definitely True
- ◯ 2
- ◯ 3
- ◯ 4
- ◯ 5
- ◯ 6
- ◯ 7 Definitely False

---

6) I cannot get addicted to opioid medication unless I enjoy taking it.
- ◯ 1 Definitely True
- ◯ 2
- ◯ 3
- ◯ 4
- ◯ 5
- ◯ 6
- ◯ 7 Definitely False

7) I cannot become addicted to opioids if my doctor prescribed them for me to take for my severe pain.

◯ 1 Definitely True
◯ 2
◯ 3
◯ 4
◯ 5
◯ 6
◯ 7 Definitely False

8) I cannot overdose or die of opioid overdose if my doctor prescribed them for me to take for my severe pain.

◯ 1 Definitely True
◯ 2
◯ 3
◯ 4
◯ 5
◯ 6
◯ 7 Definitely False

PROMIS SF v1.1 - Pain Interference 6b

Please complete the survey below.

Thank you!

FINAL RESULTS

T-score ______________________

Standard Error ______________________

PAININ3

In the past 7 days
How much did pain interfere with your enjoyment of life?

- ◯ Not at all
- ◯ A little bit
- ◯ Somewhat
- ◯ Quite a bit
- ◯ Very much

PAININ9

In the past 7 days
How much did pain interfere with your day to day activities?

- ◯ Not at all
- ◯ A little bit
- ◯ Somewhat
- ◯ Quite a bit
- ◯ Very much

PAININ8

In the past 7 days
How much did pain interfere with your ability to concentrate?

- ◯ Not at all
- ◯ A little bit
- ◯ Somewhat
- ◯ Quite a bit
- ◯ Very much

PAININ10

In the past 7 days
How much did pain interfere with your enjoyment of recreational activities?

◯ Not at all
◯ A little bit
◯ Somewhat
◯ Quite a bit
◯ Very much

PAININ14

In the past 7 days
How much did pain interfere with doing your tasks away from home (e.g., getting groceries, running errands)?

- ◯ Not at all
- ◯ A little bit
- ◯ Somewhat
- ◯ Quite a bit
- ◯ Very much

PAININ26

In the past 7 days
How often did pain keep you from socializing with others?

◯ Never
◯ Rarely
◯ Sometimes
◯ Often
◯ Always

Acknowledgment: PROMIS Health Organization and Assessment Center℠ View full acknowledgment

PROMIS SF v1.0 - Sleep Disturbance 6a

Please complete the survey below.

Thank you!

FINAL RESULTS

T-score

____

Standard Error

____

Sleep116

In the past 7 days
My sleep was refreshing.

- ◯ Not at all
- ◯ A little bit
- ◯ Somewhat
- ◯ Quite a bit
- ◯ Very much

Sleep20

In the past 7 days
I had a problem with my sleep.

- ◯ Not at all
- ◯ A little bit
- ◯ Somewhat
- ◯ Quite a bit
- ◯ Very much

Sleep44

In the past 7 days
I had difficulty falling asleep.

◯ Not at all
◯ A little bit
◯ Somewhat
◯ Quite a bit
◯ Very much

Sleep108

In the past 7 days
My sleep was restless.

- ◯ Not at all
- ◯ A little bit
- ◯ Somewhat
- ◯ Quite a bit
- ◯ Very much

Sleep72

In the past 7 days
I tried hard to get to sleep.

- ◯ Not at all
- ◯ A little bit
- ◯ Somewhat
- ◯ Quite a bit
- ◯ Very much

Sleep109

In the past 7 days
My sleep quality was...

- ◯ Very poor
- ◯ Poor
- ◯ Fair
- ◯ Good
- ◯ Very good

Acknowledgment: PROMIS Health Organization and Assessment Center℠ View full acknowledgment

PROMIS SF v1.2 - Physical Function 6b

Please complete the survey below.

Thank you!

FINAL RESULTS

T-score ______________________

Standard Error ______________________

PFA11

Are you able to do chores such as vacuuming or yard work?

- ◯ Without any difficulty
- ◯ With a little difficulty
- ◯ With some difficulty
- ◯ With much difficulty
- ◯ Unable to do

PFA21

Are you able to go up and down stairs at a normal pace?

- ◯ Without any difficulty
- ◯ With a little difficulty
- ◯ With some difficulty
- ◯ With much difficulty
- ◯ Unable to do

PFA23

Are you able to go for a walk of at least 15 minutes?

- ◯ Without any difficulty
- ◯ With a little difficulty
- ◯ With some difficulty
- ◯ With much difficulty
- ◯ Unable to do

PFA53

Are you able to run errands and shop?

- ◯ Without any difficulty
- ◯ With a little difficulty
- ◯ With some difficulty
- ◯ With much difficulty
- ◯ Unable to do

PFC12

Does your health now limit you in doing two hours of physical labor?

- ◯ Not at all
- ◯ Very little
- ◯ Somewhat
- ◯ Quite a lot
- ◯ Cannot do

PFB1

Does your health now limit you in doing moderate work around the house like vacuuming, sweeping floors or carrying in groceries?	◯ Not at all<br>◯ Very little<br>◯ Somewhat<br>◯ Quite a lot<br>◯ Cannot do

Acknowledgment: PROMIS Health Organization and Assessment Center℠ View full acknowledgment

PTSD Screener

Please complete the survey below.

Thank you!

Sometimes things happen to people that are unusually or especially frightening, horrible, or traumatic. Have you ever experienced this kind of event?	◯ Yes ◯ No
In the past month, have you had nightmares about the event(s) or thought about the event(s) when you did not want to?	◯ Yes ◯ No
In the past month have you tried hard not to think about the event(s) or went out of your way to avoid situations that reminded you of the event(s)?	◯ Yes ◯ No
In the past month have you been constantly on guard, watchful, or easily startled?	◯ Yes ◯ No
In the past month have you felt numb or detached from people, activities, or your surroundings?	◯ Yes ◯ No
In the past month have you felt guilty or unable to stop blaming yourself or others for the event(s) or any problems the event(s) may have caused?	◯ Yes ◯ No
PTSD Total	________________________

PHQ-4

Please complete the survey below.

Thank you!

Over the last two weeks, how often have you been bothered by the following problems?

---

1) Feeling nervous, anxious or on edge
- ◯ Not at all
- ◯ Several days
- ◯ More than half the days
- ◯ Nearly every day

2) Not being able to stop or control worrying
- ◯ Not at all
- ◯ Several days
- ◯ More than half the days
- ◯ Nearly every day

3) Feeling down, depressed or hopeless
- ◯ Not at all
- ◯ Several days
- ◯ More than half the days
- ◯ Nearly every day

4) Little interest or pleasure in doing things
- ◯ Not at all
- ◯ Several days
- ◯ More than half the days
- ◯ Nearly every day

5) PHQ-4 Total ______________________

6) PHQ-4 Anxiety ______________________

7) PHQ-4 Depression ______________________

SAHL-E

Please complete the survey below.

Thank you!

Which of the two words is most similar to the bolded word? If you don't know the answer, please say or select "I don't know"

1)	kidney	◯ urine ◯ fever ◯ I don't know
2)	occupation	◯ work ◯ education ◯ I don't know
3)	medication	◯ instrument ◯ treatment ◯ I don't know
4)	nutrition	◯ healthy ◯ soda ◯ I don't know
5)	miscarriage	◯ loss ◯ marriage ◯ I don't know
6)	infection	◯ plant ◯ virus ◯ I don't know
7)	alcoholism	◯ addiction ◯ recreation ◯ I don't know
8)	pregnancy	◯ birth ◯ childhood ◯ I don't know
9)	seizure	◯ dizzy ◯ calm ◯ I don't know
10)	dose	◯ sleep ◯ amount ◯ I don't know
11)	hormones	◯ growth ◯ harmony ◯ I don't know
12)	abnormal	◯ different ◯ similar ◯ I don't know
13)	directed	◯ instruction ◯ decision ◯ I don't know
14)	nerves	◯ bored ◯ anxiety ◯ I don't know
15)	constipation	◯ blocked ◯ loose ◯ I don't know
16)	diagnosis	◯ evaluation ◯ recovery ◯ I don't know
17)	hemorrhoids	◯ veins ◯ heart ◯ I don't know
18)	syphilis	◯ contraception ◯ condom ◯ I don't know

PMQ/PDUQ baseline

Please complete the survey below.

Thank you!

MISC Questions

1) In the past, I have had some difficulty getting the medication I need from my doctor(s).
   - ◯ Disagree
   - ◯ Somewhat Disagree
   - ◯ Neutral
   - ◯ Somewhat Agree
   - ◯ Agree

2) I have clear preferences about the type of pain medication I need.
   - ◯ Disagree
   - ◯ Somewhat Disagree
   - ◯ Neutral
   - ◯ Somewhat Agree
   - ◯ Agree

3) It is important to me to try ways of managing my pain in addition to the medication.
   - ◯ Disagree
   - ◯ Somewhat Disagree
   - ◯ Neutral
   - ◯ Somewhat Agree
   - ◯ Agree

4) I save any unused pain medication I have in case I need it later.
   - ◯ Never
   - ◯ Occasionally
   - ◯ Sometimes
   - ◯ Often
   - ◯ Always

5) Have you ever had to buy pain medication on the street?
   - ◯ Yes
   - ◯ No

6) Have you ever borrowed pain medication from a friend or family member?
   - ◯ Yes
   - ◯ No

PMQ/PDUQ 2 weeks

Please complete the survey below.

Thank you!

1)	After you left the hospital, did you go directly to your own home, to someone else's home, or to another health facility?	◯ Own home<br>◯ Someone else's home<br>◯ Another health facility
2)	Have you visited the Emergency Department or Urgent Care since your last visit because of pain?	◯ Yes<br>◯ No
3)	If yes, which hospital?	__________________________________
4)	Are you currently taking medications for pain?	◯ Yes<br>◯ No
5)	If yes, which of the following non-opioid medications are you taking for pain?	☐ Acetaminophen (Tylenol)<br>☐ Ibuprofen (Motrin)<br>☐ Naproxen (Naprosen, Aleve)<br>☐ Gabapentin (Neurontin)<br>☐ Lidocaine patches<br>☐ Diclofenac<br>☐ None<br>☐ Other
6)	If yes, which of the following non-opioid medications are you taking for pain?	☐ Acetaminophen (Tylenol)<br>☐ Ibuprofen (Motrin)<br>☐ Naproxen (Naprosen, Aleve)<br>☐ Gabapentin (Neurontin)<br>☐ Lidocaine patches<br>☐ Diclofenac<br>☐ None<br>☐ Other
7)	If yes, which of the following OPIOID medications have you taken in the LAST WEEK?	☐ Tramadol<br>☐ Percocet<br>☐ Lortab<br>☐ Vicodin<br>☐ Hydrocodone<br>☐ Codeine<br>☐ Oxycodone<br>☐ Oxycontin<br>☐ Fentanyl<br>☐ Other<br>☐ Not Sure<br>☐ None
8)	Have you had to take more than prescribed because your pain wasn't managed?	◯ Yes<br>◯ No

9) If no, how did you dispose of your unused opioid pills?

- ☐ I used an opioid dispoal bag
- ☐ I took my pills to a disposal site
- ☐ I still have the pills/I am saving the pills
- ☐ I ran out and don't have any pills left
- ☐ I gave it to friends, family, or someone I know
- ☐ I flushed it
- ☐ Other

10) If you still have your opioid prescription, why?

____

11) How have you obtained your opioid medications? (Select all that apply)

- ☐ Prescribed by a doctor for pain
- ☐ Borrowed from family members or friends
- ☐ Bought on the street (other than in the pharmacy)
- ☐ Already had it
- ☐ Had some leftover from a previous prescription

12) In the last month, have you smoked cigarettes/vaped?

- ☐ Smoking: every day
- ☐ Smoking: some days
- ☐ Vaping: every day
- ☐ Vaping: some days
- ☐ No

13) At times, I drink alcohol to help control my pain.

- ◯ Never
- ◯ Occasionally
- ◯ Sometimes
- ◯ Often
- ◯ Always

14) Have you used any of these in the past MONTH? (check all that apply)

- ☐ Marijuana (cannabis, THC oil, dabbing)
- ☐ Synthetic marijuana (spice)
- ☐ Cocaine
- ☐ Heroin
- ☐ Hallucinogens (LSD, PCP, peyote, mescaline, psilocybin mushrooms, and Ecstasy)
- ☐ Inhalants (nitrous oxide, amyl nitrate, cleaning fluids, gasoline, spray paints, aerosol sprays, glue)
- ☐ Stimulants (meth, amphetamines, etc)
- ☐ None

15) What else have you been using to manage pain?

- ☐ Physical therapy
- ☐ Ice/heat
- ☐ Exercise
- ☐ Meditation
- ☐ Massage therapy
- ☐ Topical creams
- ☐ CBD
- ☐ LCS Interventions
- ☐ None
- ☐ Other

16) I believe I am receiving enough medication to relieve my pain.

- ◯ Disagree
- ◯ Somewhat Disagree
- ◯ Neutral
- ◯ Somewhat Agree
- ◯ Agree

17) I believe I would feel better with a higher dosage of my pain medication.
- ◯ Disagree
- ◯ Somewhat Disagree
- ◯ Neutral
- ◯ Somewhat Agree
- ◯ Agree

18) At times, I take pain medication when I feel anxious, sad, or when I need help sleeping.
- ◯ Never
- ◯ Occasionally
- ◯ Sometimes
- ◯ Often
- ◯ Always

19) I get pain medication from more than one doctor in order to have enough medication for my pain.
- ◯ Never
- ◯ Occasionally
- ◯ Sometimes
- ◯ Often
- ◯ Always

20) Date of participant's surgery ______________________

21) Date participant completed surveys ______________________

22) Days since surgery ______________________

PMQ/PDUQ 6 weeks

Please complete the survey below.

Thank you!

1)	Have you visited the Emergency Department or Urgent Care since your last visit because of pain?	◯ Yes<br>◯ No
2)	If yes, which hospital?	__________________________________
3)	Are you currently taking medications for pain?	◯ Yes<br>◯ No
4)	If yes, which of the following non-opioid medications are you taking for pain?	☐ Acetaminophen (Tylenol)<br>☐ Ibuprofen (Motrin)<br>☐ Naproxen (Naprosen, Aleve)<br>☐ Gabapentin (Neurontin)<br>☐ Lidocaine patches<br>☐ Diclofenac<br>☐ None<br>☐ Other
5)	If yes, which of the following non-opioid medications are you taking for pain?	☐ Acetaminophen (Tylenol)<br>☐ Ibuprofen (Motrin)<br>☐ Naproxen (Naprosen, Aleve)<br>☐ Gabapentin (Neurontin)<br>☐ Lidocaine patches<br>☐ Diclofenac<br>☐ None<br>☐ Other
6)	If yes, which of the following OPIOID medications have you taken in the LAST WEEK?	☐ Tramadol<br>☐ Percocet<br>☐ Lortab<br>☐ Vicodin<br>☐ Hydrocodone<br>☐ Codeine<br>☐ Oxycodone<br>☐ Oxycontin<br>☐ Fentanyl<br>☐ Other<br>☐ Not Sure<br>☐ None
7)	Have you had to take more than prescribed because your pain wasn't managed?	◯ Yes<br>◯ No
8)	If no, how did you dispose of your unused opioid pills?	☐ I used an opioid dispoal bag<br>☐ I took my pills to a disposal site<br>☐ I still have the pills/I am saving the pills<br>☐ I ran out and don't have any pills left<br>☐ I gave it to friends, family, or someone I know<br>☐ I flushed it<br>☐ Other
9)	If you still have your opioid prescription, why?	__________________________________

10) How have you obtained your opioid medications? (Select all that apply)

- ☐ Prescribed by a doctor for pain
- ☐ Borrowed from family members or friends
- ☐ Bought on the street (other than in the pharmacy)
- ☐ Already had it
- ☐ Had some leftover from a previous prescription

11) In the last month, have you smoked cigarettes/vaped?

- ☐ Smoking: every day
- ☐ Smoking: some days
- ☐ Vaping: every day
- ☐ Vaping: some days
- ☐ No

12) Have you used alcohol to numb or treat your pain?

- ◯ Yes
- ◯ No

13) Have you used any of these in the past MONTH? (check all that apply)

- ☐ Marijuana (cannabis, THC oil, dabbing)
- ☐ Synthetic marijuana (spice)
- ☐ Cocaine
- ☐ Heroin
- ☐ Hallucinogens (LSD, PCP, peyote, mescaline, psilocybin mushrooms, and Ecstasy)
- ☐ Inhalants (nitrous oxide, amyl nitrate, cleaning fluids, gasoline, spray paints, aerosol sprays, glue)
- ☐ Stimulants (meth, amphetamines, etc)
- ☐ None

14) What else have you been using to manage pain?

- ☐ Physical therapy
- ☐ Ice/heat
- ☐ Exercise
- ☐ Meditation
- ☐ Massage therapy
- ☐ Topical creams
- ☐ CBD
- ☐ LCS Interventions
- ☐ None
- ☐ Other

15) Family members seem to think that I may be too dependent on my pain medication.

- ◯ Disagree
- ◯ Somewhat Disagree
- ◯ Neutral
- ◯ Somewhat Agree
- ◯ Agree

16) I get pain medication from more than one doctor in order to have enough medication for my pain.

- ◯ Never
- ◯ Occasionally
- ◯ Sometimes
- ◯ Often
- ◯ Always

17) At times I think I may be too dependent on pain medication.

- ◯ Never
- ◯ Occasionally
- ◯ Sometimes
- ◯ Often
- ◯ Always

18) To help me out, family members have obtained pain medications for me from their own doctors.
- ◯ Never
- ◯ Occasionally
- ◯ Sometimes
- ◯ Often
- ◯ Always

19) I find it helpful to call my doctor or clinic to talk about how my pain medication is working.
- ◯ Never
- ◯ Occasionally
- ◯ Sometimes
- ◯ Often
- ◯ Always

20) Have you had to call in for more pain medications because your prescription ran out?
- ◯ Yes
- ◯ No

21) Date of participant's surgery

____

22) Date participant completed surveys

____

23) days since surgery

____

PMQ/PDUQ 12 weeks

Please complete the survey below.

Thank you!

---

1) Have you visited the Emergency Department or Urgent Care since your last visit because of pain?
- ◯ Yes
- ◯ No

2) If yes, which hospital?

____

3) Are you currently taking medications for pain?
- ◯ Yes
- ◯ No

4) If yes, which of the following non-opioid medications are you taking for pain?
- ☐ Acetaminophen (Tylenol)
- ☐ Ibuprofen (Motrin)
- ☐ Naproxen (Naprosen, Aleve)
- ☐ Gabapentin (Neurontin)
- ☐ Lidocaine patches
- ☐ Diclofenac
- ☐ None
- ☐ Other

5) If yes, which of the following non-opioid medications are you taking for pain?
- ☐ Acetaminophen (Tylenol)
- ☐ Ibuprofen (Motrin)
- ☐ Naproxen (Naprosen, Aleve)
- ☐ Gabapentin (Neurontin)
- ☐ Lidocaine patches
- ☐ Diclofenac
- ☐ None
- ☐ Other

6) If yes, which of the following OPIOID medications have you taken in the LAST WEEK?
- ☐ Tramadol
- ☐ Percocet
- ☐ Lortab
- ☐ Vicodin
- ☐ Hydrocodone
- ☐ Codeine
- ☐ Oxycodone
- ☐ Oxycontin
- ☐ Fentanyl
- ☐ Other
- ☐ Not Sure
- ☐ None

7) Have you had to take more than prescribed because your pain wasn't managed?
- ◯ Yes
- ◯ No

8) If no, how did you dispose of your unused opioid pills?
- ☐ I used an opioid dispoal bag
- ☐ I took my pills to a disposal site
- ☐ I still have the pills/I am saving the pills
- ☐ I ran out and don't have any pills left
- ☐ I gave it to friends, family, or someone I know
- ☐ I flushed it
- ☐ Other

9) If you still have your opioid prescription, why?

____

10)	How have you obtained your opioid medications? (Select all that apply)	☐ Prescribed by a doctor for pain<br>☐ Borrowed from family members or friends<br>☐ Bought on the street (other than in the pharmacy)<br>☐ Already had it<br>☐ Had some leftover from a previous prescription
11)	In the last month, have you smoked cigarettes/vaped?	☐ Smoking: every day<br>☐ Smoking: some days<br>☐ Vaping: every day<br>☐ Vaping: some days<br>☐ No
12)	Have you used alcohol to numb or treat your pain?	◯ Yes<br>◯ No
13)	Have you used any of these in the past MONTH? (check all that apply)	☐ Marijuana (cannabis, THC oil, dabbing)<br>☐ Synthetic marijuana (spice)<br>☐ Cocaine<br>☐ Heroin<br>☐ Hallucinogens (LSD, PCP, peyote, mescaline, psilocybin mushrooms, and Ecstasy)<br>☐ Inhalants (nitrous oxide, amyl nitrate, cleaning fluids, gasoline, spray paints, aerosol sprays, glue)<br>☐ Stimulants (meth, amphetamines, etc)<br>☐ None
14)	What else have you been using to manage pain?	☐ Physical therapy<br>☐ Ice/heat<br>☐ Exercise<br>☐ Meditation<br>☐ Massage therapy<br>☐ Topical creams<br>☐ CBD<br>☐ LCS Interventions<br>☐ None<br>☐ Other
15)	How many times in the past year have you asked your doctor to increase your prescription dosage of pain medication in order to get relief?	◯ Never<br>◯ 1 Time<br>◯ 2 Times<br>◯ 3 Times<br>◯ 4+ Times
16)	How many times in the past year have you run out of pain medication early and had to request an early refill?	◯ Never<br>◯ 1 Time<br>◯ 2 Times<br>◯ 3 Times<br>◯ 4+ Times
17)	How many times in the past year have you accidentally misplaced your prescription for pain medication and had to ask for another?	◯ Never<br>◯ 1 Time<br>◯ 2 Times<br>◯ 3 Times<br>◯ 4+ Times
18)	Has your pain been adequately treated over the past 6 months?	◯ Yes<br>◯ No

19) Have you been given pain medications from more than one clinic over the last 6 months?
◯ Yes
◯ No

20) Have you ever had to buy pain medications on the street?
◯ Yes
◯ No

21) Have you ever borrowed pain medications from a friend or family member?
◯ Yes
◯ No

22) Have those in your family or among your friends ever obtained pain medications for you?
◯ Yes
◯ No

23) Date of participant's surgery
____

24) Date participant completed survey
____

25) Days since surgery
____

Pain Numeric Rating Scale

Please complete the survey below.

Thank you!

---

1) Over the past 24-hours how would you rate your pain, on average, from 0, no pain, to 10, the worst pain imaginable?

◯ 0
◯ 1
◯ 2
◯ 3
◯ 4
◯ 5
◯ 6
◯ 7
◯ 8
◯ 9
◯ 10

Discharge Data

Please complete the survey below.

Thank you!

1) Inpatient morphine milligram equivalents ______________________

2) last pain score prior to discharge ______________________

3) total discharge morphine milligram equivalents (dosage X number of pills) ______________________

4) days worth of medication ______________________

5) MME/Day ______________________

6) Length of stay, in days ______________________

7) Discharge status
   - ◯ home
   - ◯ facility
   - ◯ prison
   - ◯ expired
   - ◯ other

Patient Satisfaction Survey

Please complete the survey below.

Thank you!

---

1) During my hospital stay, my healthcare team helped manage my stress or anxiety.

- ◯ Strongly agree
- ◯ Agree
- ◯ Somewhat agree
- ◯ Neither agree nor disagree
- ◯ Somewhat disagree
- ◯ Disagree

---

2) When I left the hospital, I understood the risks and side effects of my opioid pain medication.

- ◯ Strongly agree
- ◯ Agree
- ◯ Somewhat agree
- ◯ Neither agree nor disagree
- ◯ Somewhat disagree
- ◯ Disagree
- ◯ Strongly disagree

---

3) My healthcare team was committed to my wellbeing.

- ◯ Strongly agree
- ◯ Agree
- ◯ Somewhat agree
- ◯ Neither agree nor disagree
- ◯ Somewhat disagree
- ◯ Disagree
- ◯ Strongly disagree

---

4) I received resources for after my stay.

- ◯ Strongly agree
- ◯ Agree
- ◯ Somewhat agree
- ◯ Neither agree nor disagree
- ◯ Somewhat disagree
- ◯ Disagree
- ◯ Strongly disagree

---

5) I felt supported by my healthcare team when I could not have friends or family visit.

- ◯ Strongly agree
- ◯ Agree
- ◯ Somewhat agree
- ◯ Neither agree nor disagree
- ◯ Somewhat disagree
- ◯ Disagree
- ◯ Strongly disagree

---

6) I was educated on what Narcan is and why my doctor might prescribe it.

- ◯ Yes
- ◯ No
- ◯ I was educated on what Narcan is, but not why my doctor might prescribe it
- ◯ Not Sure

---

7) When I left the hospital, I had a good understanding of the things I was responsible for in managing my health.

- ◯ Strongly Disagree
- ◯ Disagree
- ◯ Agree
- ◯ Strongly Agree
- ◯ N/A

8) When I left the hospital, I clearly understood the purpose for taking each of my medications.
◯ Strongly Disagree
◯ Disagree
◯ Agree
◯ Strongly Agree
◯ N/A

9) During this hospital stay were you given any medication you had not taken before?
◯ Yes
◯ No

10) If yes, Before giving you any new medicine, how often did hospital staff tell you what the medicine was for?
◯ Never
◯ Sometimes
◯ Usually
◯ Always

11) If yes, Before giving you any new medicine, how often did hospital staff describe possible side effects in a way you could understand?
◯ Never
◯ Sometimes
◯ Usually
◯ Always

12) During this hospital stay, did doctors, nurses, or other hospital staff talk with you about whether you would have the help you needed when you left the hospital?
◯ Yes
◯ No
◯ N/A

13) During this hospital stay, did you get information in writing about what symptoms or health problems to look out for after you left the hospital?
◯ Yes
◯ No
◯ N/A

14) Using any number from 0 to 10, where 0 is the worst hospital possible and 10 is the best hospital possible, what number would you use to rate this hospital during your stay?
◯ 0
◯ 1
◯ 2
◯ 3
◯ 4
◯ 5
◯ 6
◯ 7
◯ 8
◯ 9
◯ 10

15) During this hospital stay, staff took my preferences and those of my family or caregiver into account in deciding what my health care needs would be when I left.
◯ Strongly Disagree
◯ Disagree
◯ Agree
◯ Strongly Agree

Naloxone Questionnaire

Please complete the survey below.

Thank you!

Do you feel confident using Narcan?	◯ Yes<br>◯ No<br>◯ Unsure
Did you fill your Narcan prescription ?	◯ Yes<br>◯ No
If you did fill your prescription, how much did it cost you?	__________________________________
Did you use your Narcan prescription or any Narcan since we last saw you?	◯ Yes<br>◯ No
If yes, can you provide any details about your Narcan use?	◯ Used for myself<br>◯ Used for a friends<br>◯ Used for a family<br>◯ Gave it away<br>◯ Other

Inpatient Medications

Please complete the survey below.

Thank you!

Pain Medications - Non Opioids	☐ Ativan (Benzodiazepines)<br>☐ Midazolam (Benzodiazepines)<br>☐ Diazepam (Benzodiazepines)<br>☐ Clonazepam (Benzodiazepines)<br>☐ Lidocaine Patch (Local)<br>☐ Epidural Infusion (Local)<br>☐ Continuous Peripheral Nerve Block (Local)<br>☐ Clonidine Patch (alpha-2-agonist)<br>☐ Dexmedetomidine (alpha-2-agonist)<br>☐ Tizanidine (alpha-2-agonist)<br>☐ Gabapentin (anticonvulsant)<br>☐ Pregabalin (anticonvulsant)<br>☐ Topomax (anticonvulsant)<br>☐ Pamelor (antidepressant)<br>☐ Elavil (antidepressant)<br>☐ Tylenol<br>☐ TCA<br>☐ Effexor (SNRI)<br>☐ celebrex (NSAID)<br>☐ toradol (NSAID)<br>☐ mobic (NSAID)<br>☐ motrin (NSAID)<br>☐ aspirin (NSAID)
Pain Medication - Opioids	☐ Hydromorphone (Dilaudid)<br>☐ Hydromorphone (Dilaudid) PCA<br>☐ Morphine IR (MSIR)<br>☐ Morphine SR (MS contin)<br>☐ Oxycodone SR (Oxycotin)<br>☐ Oxycodone IR (Oxy IR)<br>☐ Oxycodone/Acetaminophen (Percocet)<br>☐ Methadone (Dolophine/Methadose)<br>☐ Fentanyl<br>☐ Meperidine (Demerol)<br>☐ Oxymorphone (Opana)<br>☐ Other
Hydromorphone (Dilaudid) - Dose	__________________________
Hydromorphone (Dilaudid) - Route	○ IV<br>○ Oral
Hydromorphone (Dilaudid) PCA - Dose	__________________________
Morphine IR - Dose	__________________________
Morphine IR - Route	○ IV<br>○ Oral
Morphine SR - Dose	__________________________

Morphine SR - Route	◯ IV<br>◯ Oral
Oxycodone SR (oxycotin) - Dose	________________
Oxycodone SR (oxycotin) - Route	◯ IV<br>◯ Oral
Oxycodone IR - Dose	________________
Oxycodone IR - Route	◯ IV<br>◯ Oral
Oxycodone / Acetaminophen (Percocet) - Dose	________________
Oxycodone / Acetaminophen (Percocet) - Route	◯ IV<br>◯ Oral
Methadone - Dose	________________
Methadone - Route	◯ IV<br>◯ Oral
Fentanyl - Dose	________________
Fentanyl - Route	◯ IV<br>◯ Oral<br>◯ Patch
Merpredine - Dose	________________
Merpredine - Route	◯ IV<br>◯ Oral
Oxymorphone - Dose	________________
Oxymorphone - Route	◯ IV<br>◯ Oral
Other - Name of medication	________________
Other - Dose	________________
Other - Route	◯ IV<br>◯ Oral

Research Team Facing Intervention Survey

Please complete the survey below.

Thank you!

Did the participant meet with the LCS	◯ Yes<br>◯ No
The Revised ORT score for this patient [ort_score]	__________________________________
LCS Name	____________________________
LCS Intervention Date and Time START	____________________________
LCS Intervention Date and Time END	____________________________
Duration of Intervention	____________________________
How many times did the LCS meet with the participant during their hospitalization	____________________________
Was the participant trained on PROGRESSIVE MUSCLE RELAXATION approaches?	◯ Yes<br>◯ No
Was the participant trained on SOUND THERAPY approaches ?	◯ Yes<br>◯ No
Was the participant trained on DIAPHRAGMATIC BREATHING approaches ?	◯ Yes<br>◯ No
Was the participant trained on TRACKING approaches ?	◯ Yes<br>◯ No
Was the participant trained on RESOURCING approaches ?	◯ Yes<br>◯ No
Was the participant trained on GROUNDING approaches ?	◯ Yes<br>◯ No
Was the participant trained on GESTURING approaches ?	◯ Yes<br>◯ No
Was the participant trained on SHIFT AND STAY approaches ?	◯ Yes<br>◯ No
Was the participant trained on HELP NOW! approaches ?	◯ Yes<br>◯ No

Was the participant trained on SLEEP HYGIENE approaches ?	◯ Yes<br>◯ No
Did the LCS use MOTIVATIONAL INTERVIEWING approaches ?	◯ Yes<br>◯ No
Did the LCS provide DISTRACTION MATERIALS ?	◯ Yes<br>◯ No
Did the LCS provide a BLANKET ?	◯ Yes<br>◯ No
If Help Now! was used which skills did the LCS teach?	☐ TBD 1<br>☐ TBD 2<br>☐ TBD 3
If resourcing was used, what resource did the patient self identify?	__________________________________
If the patient got distraction materials from the LCS what were they?	__________________________________
If the LCS showed the iChill app to the patient did they download it?	◯ Yes<br>◯ No
Did the patient have prior knowledge of Narcan?	◯ Yes<br>◯ No
Patient reports past opioid misuse	◯ Yes<br>◯ No
Patient reports having taken opioids before	◯ Yes<br>◯ No
Patient received education on:	☐ Opioid disposal education<br>☐ Opioid tapering education<br>☐ Opioid common side effects<br>☐ What not to mix with opioids<br>☐ Patient did not want education on opioid safety
Patient received narcan education on:	☐ Explained what Narcan is<br>☐ Explained how to administer Narcan<br>☐ Presentation of Resource Guide<br>☐ Did not want narcan education<br>☐ Already knew about narcan and did not want more education

Patient referred to:	☐ Psychiatry<br>☐ Social work<br>☐ Acute pain service<br>☐ Other<br>☐ PTSD (The Grady Trauma Project)<br>☐ OUD Addiction medicine (Dr. Stek and team)<br>☐ Food Insecurity (Food as Medicine project)<br>☐ Homelessness (Social Work)<br>☐ Suicide Ideation (Grady NIA project)<br>☐ Alcohol Use Disorder (Dr. Heiman, Primary Care)<br>☐ Mental Health/ Substance Use (Behavioral health outpatient services)<br>☐ Smoking Cessation (Freedom From Smoking Program)
If referred to "other" who:	______________________________
Other notes or comments	_____________________________________

Patient Facing Intervention Survey

Please complete the survey below.

Thank you!

Question	Options
Which Life Care Specialist did you meet with?	◯ Bailey<br>◯ Lauren<br>◯ Carter<br>◯ Anna<br>◯ Other<br>◯ Not sure<br>◯ I did not meet with a Life Care Specialist
Please let us know if you agree or disagree with the statements below:	
My interaction with the Life Care Specialist (LCS) was helpful in managing my pain	◯ Agree<br>◯ Neutral<br>◯ Disagree
The resources and training provided to me by the LCS were helpful	◯ Agree<br>◯ Neutral<br>◯ Disagree
During this hospital stay, the education I received help me understand the risks and side effects of the pain mediation I was discharged with	◯ Agree<br>◯ Neutral<br>◯ Disagree
The LCS was a valuable addition to my healthcare team	◯ Agree<br>◯ Neutral<br>◯ Disagree
My interaction with the LCS helped reduce my need for opioid pain medication at home	◯ Agree<br>◯ Neutral<br>◯ Disagree
The resources provided to me by the LCS were easy to understand	◯ Agree<br>◯ Neutral<br>◯ Disagree
The Pain Plan instructions were easy to understand	◯ Agree<br>◯ Neutral<br>◯ Disagree
Which of the following approaches did you use to help manage your pain:	☐ Relaxed Breathing<br>☐ Progressive Muscle Relaxation<br>☐ Guided Imagery<br>☐ Meditation<br>☐ Mindfulness<br>☐ 6 Ranges of Motion<br>☐ Essential Oils<br>☐ Music Therapy<br>☐ Vibra Cool/Buzzy
Did you find relaxed breathing to be helpful for managing your pain?	◯ Yes<br>◯ No<br>◯ Unsure

Did you find progressive muscle relaxation to be helpful for managing your pain?	◯ Yes<br>◯ No<br>◯ Unsure
Did you find guided imagery to be helpful for managing your pain?	◯ Yes<br>◯ No<br>◯ Unsure
Did you find meditation to be helpful for managing your pain?	◯ Yes<br>◯ No<br>◯ Unsure
Did you find mindfulness to be helpful for managing your pain?	◯ Yes<br>◯ No<br>◯ Unsure
Did you find 6 ranges of motion to be helpful for managing your pain?	◯ Yes<br>◯ No<br>◯ Unsure
Did you find essential oils to be helpful for managing your pain?	◯ Yes<br>◯ No<br>◯ Unsure
Did you find music therapy to be helpful for managing your pain?	◯ Yes<br>◯ No<br>◯ Unsure
Did you find vibra cool to be helpful for managing your pain?	◯ Yes<br>◯ No<br>◯ Unsure
Did you return the unused portion of your pain medication at your follow up visit?	◯ Yes<br>◯ No

Trauma Registry Data

Please complete the survey below.

Thank you!

Arrived From	◯ Scene<br>◯ Referring Hospital<br>◯ Other
DOB	__________________________________
Age	__________________________________
Gender	◯ Male<br>◯ Female
Race	◯ Asian<br>◯ Native Hawaiian or Other Pacific Islander<br>◯ Other Race<br>◯ American Indian<br>◯ Black or African American<br>◯ White
Ethnicity	◯ Hispanic or Latino<br>◯ Not Hispanic or Latino
Injury Date	__________________________________
Place of Injury	◯ Street<br>◯ Residential Institution<br>◯ Home<br>◯ Industry<br>◯ Public Building<br>◯ Recreation<br>◯ Unspecified
Chief Complaint	__________________________________
Work Related	◯ No<br>◯ Yes
Report of Physical Abuse	◯ No<br>◯ Yes
Primary E-Code	__________________________________
Secondary E-Code	__________________________________
Injury Type	◯ Blunt<br>◯ Penetrating

Direct Admit?	◯ No<br>◯ Yes
Admitting service	◯ Orthopaedics<br>◯ Trauma<br>◯ Other Non-Surgical
ED Arrival	__________________________________
Ed Departure	__________________________________
Time in ED	__________________________________
Mode of Arrival	◯ Ground Ambulance<br>◯ Private/Public Vehicle/Walk-In
Response Activation Level	◯ No Trauma Activation<br>◯ Consult<br>◯ Partial<br>◯ Full
ED Disposition	◯ Operating Room<br>◯ Floor Bed (General Admission, Non-Specialty Unit Bed)<br>◯ Intensive Care Unit (ICU)
GCS: Total	__________________________________
Height	__________________________________
Weight	__________________________________
Toxicology: ETOH use Indicator	◯ No<br>◯ Yes
Toxicology: ETOH/BAC Level	__________________________________
Toxicology: Drug use Indicator	◯ No (Not Tested)<br>◯ Yes (Confirmed by Test[Illegal Use Drug])

Toxicology: Tox Screen Results	☐ AMP (Amphetamine)<br>☐ BAR (Barbiturate)<br>☐ BZO (Benzodiazepines)<br>☐ COC (Cocaine)<br>☐ mAMP (Methamphetamine)<br>☐ MDMA (Ecstasy)<br>☐ MTD (Methadone)<br>☐ OPI (Opioid)<br>☐ OXY (Oxycodone)<br>☐ PCP (Phencyclidine)<br>☐ TCA (Tricyclic Antidepressant)<br>☐ THC (Cannabinoid)<br>☐ Other
Injury #1(Text)	______________________
Injury #1(Code)	______________________
Injury #2 (Text)	______________________
Inury #2 (Code)	______________________
Injury #3 (Text)	______________________
Injury #3 (Code)	______________________
ISS	______________________
Alcohol use Disorder	◯ No<br>◯ Yes
Anticoagulant Therapy	◯ No<br>◯ Yes
Bleeding Disorder	◯ No<br>◯ Yes
Currently Receiving Chemo for Cancer	◯ No<br>◯ Yes
Congenital Anomalies	◯ No<br>◯ Yes
Congestive Heart Failure	◯ No<br>◯ Yes
Current Smoker	◯ No<br>◯ Yes

Chronic Renal Failure	◯ No<br>◯ Yes
Cerebrovascular Accident (CVA)	◯ No<br>◯ Yes
Diabetes	◯ No<br>◯ Yes
Disseminated Cancer	◯ No<br>◯ Yes
Advanced Directive Limiting Care	◯ No<br>◯ Yes
Functionally Dependent Health Status	◯ No<br>◯ Yes
Hx of Angina w/in 30 Days	◯ No<br>◯ Yes
Hx of Myocardial Infarction	◯ No<br>◯ Yes
Hx of Peripheral Vascular Disease	◯ No<br>◯ Yes
Hypertension Requiring Medication	◯ No<br>◯ Yes
Prematurity	◯ No<br>◯ Yes
Chronic Obstructive Pulmonary Disease	◯ No<br>◯ Yes
Steroid Use	◯ No<br>◯ Yes
Cirrhosis	◯ No<br>◯ Yes
Dementia	◯ No<br>◯ Yes
Major Psychiatric Illness	◯ No<br>◯ Yes
ADD/ADHD	◯ No<br>◯ Yes
Drug use Disorder	◯ No<br>◯ Yes

Acute Kidney Injury	◯ No<br>◯ Yes
Acute Lung Injury (ARDS)	◯ No<br>◯ Yes
Cardiac Arrest w/CPR	◯ No<br>◯ Yes
Catheter Related Blood Stream Infection	◯ No<br>◯ Yes
Decubitus Ulcer	◯ No<br>◯ Yes
Deep Surgical Site Infection	◯ No<br>◯ Yes
Drug/ETOH	◯ No<br>◯ Yes
DVT/Thrombophlebitis	◯ No<br>◯ Yes
Extremity Compartment Syndrome	◯ No<br>◯ Yes
Graft/Prosthesis/Flap Failure	◯ No<br>◯ Yes
Myocardial Infarction	◯ No<br>◯ Yes
Organ Spacer Surgical Site Infection	◯ No<br>◯ Yes
Osteomyelitis	◯ No<br>◯ Yes
Pneumonia	◯ No<br>◯ Yes
Pulmonary Embolism	◯ No<br>◯ Yes
Sepsis	◯ No<br>◯ Yes
Stroke/CVA	◯ No<br>◯ Yes
Superficial Surgical Site Infection	◯ No<br>◯ Yes

Unplanned Intubation	◯ No<br>◯ Yes
Unplanned Return to ICU	◯ No<br>◯ Yes
Unplanned Return to OR	◯ No<br>◯ Yes
Urinary Tract Infection	◯ No<br>◯ Yes
Total ICU days	__________________________
Total Vent Days	__________________________
Total Hospital Days	__________________________
Discharge Date	__________________________
Discharge To	◯ Discharged/Transferred to a short-term general hospital for inpatient care<br>◯ Discharged/Transferred to an Intermediate Care Facility (ICF)<br>◯ Discharged/Transferred to home under care of organized home health service<br>◯ Left against medical advice or discontinued care<br>◯ Deceased/Expired<br>◯ Discharged Home with No Home Services<br>◯ Discharged/Transferred to Skilled Nursing Facility (SNF)<br>◯ Discharged/Transferred to hospice care<br>◯ Discharged/Transferred to court/law enforcement<br>◯ Discharged/Transferred to inpatient rehab or designated unit<br>◯ Discharged/Transferred to Long Term Care Hospital (LTCH)<br>◯ Discharged/Transferred to a psychiatric hospital or psychiatric distinct part unit of a hospital<br>◯ Discharged/Transferred to another type of institution not defined elsewhere
Injury address zip code	__________________________
Patient address zip code	__________________________
Payor	__________________________

Patient occupation	◯ Business and Financial Operations Occupations<br>◯ Architecture and Engineering Occupations<br>◯ Community and Social Services Occupations<br>◯ Education, Training, and Library Occupations<br>◯ Healthcare Practitioners and Technical Occupations<br>◯ Protective Service Occupations<br>◯ Building and Grounds Cleaning and Maintenance<br>◯ Sales and Related Occupations<br>◯ Farming, Fishing, and Forestry Occupations<br>◯ Installation, Maintenance, and Repair Occupations<br>◯ Transportation and Material Moving Occupations<br>◯ Management Occupations<br>◯ Computer and Mathematical Occupations<br>◯ Life, Physical, and Social Science Occupations<br>◯ Legal Occupations<br>◯ Arts, Design, Entertainment, Sports, and Media<br>◯ Healthcare Support Occupations<br>◯ Food Preparation and Serving Related<br>◯ Personal Care and Service Occupations<br>◯ Office and Administrative Support Occupations<br>◯ Construction and Extraction Occupations<br>◯ Production Occupations<br>◯ Military Specific Occupations
Patient job industry	◯ Finance, Insurance, and Real Estate<br>◯ Manufacturing<br>◯ Retail Trade<br>◯ Transportation and Public Utilities<br>◯ Agriculture, Forestry, Fishing<br>◯ Professional and Business Services<br>◯ Education and Health Services<br>◯ Construction<br>◯ Government<br>◯ Natural Resources and Mining<br>◯ Information Services<br>◯ Wholesale Trade<br>◯ Leisure and Hospitality<br>◯ Leisure and Hospitality
Comorb - pregnancy	◯ No<br>◯ Yes
Readmission dates	______________________________
Investigation of physical abuse	◯ No<br>◯ Yes
BMI	______________________________
Self-inflicted	◯ No<br>◯ Yes
TBI	◯ No<br>◯ Yes

Pain Control After Discharge:

- You have been prescribed an Opioid Pain Medication and we have created pain management program that will help treat your pain, while limiting the bad side effects of the pain medication
- The goal is to treat your pain, and then quickly get you back on the road to recovery without the need for opioid pain medications
- Our expectation is that you will not need a refill of your medication prior to your clinic appointment.
- **YOU SHOULD ONLY TAKE YOUR PAIN MEDICATION IF YOU ARE HAVING PAIN.**
- Depending on the type of injury that you have - as well as other factors like medical disease and medication allergies - you will be prescribed a Pain Protocol when you are discharged from the hospital that is designed just for you. The clinical team taking care of you will discuss your individualized plan with you before you go home. This may include an opioid or opioid-like pain medication, like Percocet, oxycodone, Roxicodone, or tramadol, sometimes with prescription strength Tylenol (acetaminophen) and ibuprofen. If you are prescribed Tylenol or ibuprofen, these are scheduled doses. This means that they can be taken every few hours on a schedule - rather than an as needed basis. This may help stay ahead of your pain by treating some of the inflammation present - offering more consistent pain relief. For opioids, we will provide a personalized plan that will gradually and safely reduce the amount you need in the first few weeks after surgery.
- At any point in the process, we may engage additional pain specialists for help - including Life Care Specialists, Pain Management Physicians, or Substance Abuse Physicians or Counselors. They will help get you back on the road to recovery, even if you have battled issues like chronic pain or substance abuse in the past.

What to Know About Opioid Pain Medications?

- Sometimes patients are given opioids to control pain from injuries or after surgery. This is called acute pain. At other times, opioids are given for chronic pain. This is pain that lasts more than 3 months.
- Your provider wants you to have this fact sheet because you are taking narcotic (opioid) pain medicine. New research shows that **opioids can be life-threatening**. Knowing the following facts will limit unintended harm.

What are opioids?

- Opioids are strong prescription medicines that are used to manage very bad pain.

What do we know?
Using opioids can:

- Make you feel very sleepy, cause you to have less clear thinking, and a worse mood;
- Cause irregular menstrual periods, problems getting erections, cause weak bones, weight gain, and constipation;
- Cause your body to get used to the medication (dependence) if you take it for more than a few days. This means you could go through withdrawal when you stop taking it all of a sudden;
- Cause you to be addicted, even if you follow your doctor's instructions;
- Stop your breathing, which can lead to death.

What can help?

- Take the opioid exactly as your doctor tells you—for the shortest time and lowest dose possible and only for very bad pain.
- Do not take more medication on your own. If your pain does not improve, call your doctor.
- Do not cut, break, chew, or crush the pills unless your doctor says it is okay.
- Look at other ways to control your pain. Using ice, heat, wraps, using other non-opioid medications, stress management, deep breathing, and exercises may help.

How to stay safe?

1. **Do not share your opioid medication**. Always keep them locked up.
2. **Do not drive or operate machinery** until you know how the opioid will affect you.
3. **Do not drink alcohol or take other drugs**. Even prescriptions like **benzodiazepines** (Valium, Klonopin, or Xanax), **muscle relaxants** (Soma, Zanaflex, or Flexeril), and **sleeping pills** (Ambien, Lunesta) can cause death if taken with opioids.
4. **Only use one doctor to write your opioid prescriptions**. Usually this will be your primary care provider. Emergency Department visits and walk-in appointments are not safe places for managing chronic pain.
5. **Focus on the whole person** – your mind and body. This means understanding what is important to you as well as the different things that affect your pain. Then, you and your health care team can work together towards realistic goals.
6. Georgia law and guidelines for patients on long term opioids require patients to **sign a Consent for Long Term Opioid Therapy**. Patients are also required to have frequent urine drug tests.
7. If you have to be on high dose opioids, ask your provider about **Naloxone**. This is a medication that your family can give you if opioid makes you stop breathing.

   Depending on how much opioid medication you receive when you are discharged from the hospital, you may receive a prescription for this. Please fill this prescription, and please watch this youtube video with your family members as soon as you are discharged. We sincerely hope that you will not need this, but if you do, it could save your life

   https://www.youtube.com/watch?v=hGVSaO1oxpg

   What can I do with opioid pills I do not use?

   ∑ The US Food and Drug Administration has multiple sites across the country that can take unused pills. We strongly recommend disposing of them in this way - so that your family stays safe ·

   https://www.fda.gov/drugs/disposal-unused-medicines-what-you-should-know/drug-disposal-drug-take-back-locations

   Please know that narcotic medication cannot be called in to a pharmacy. Prescriptions have to be hand signed and given as a hard paper copy.

You Are Being Asked to Be in a Research Study

Concise presentation of key concepts

You are being asked to be in a research study. A research study is designed to answer a scientific question. If you agree to be in the study you will be one of 500 people who are being studied, at Emory.

Why is this study being done?
This study is being done to learn more about Life Care Specialist (LCS) position and the value it provides to the patient and the healthcare setting in reducing misuse of prescriptions. Additionally, the resources required to carry it through. You are being asked to be in this research study because you have had or will be having surgery with one of our Orthopaedic surgeons.

Do you have to be in the study?
It is your decision to be part of this research study. You do not have to be in it. Your choice will not affect your access to medical care for your condition. Before you make your decision, you should take time to learn about the study.

What do I have to do if I choose to participate in this study?
If you are eligible and want to be a part of the study, you have an equal chance of being assigned to any one of the two groups.

If you are in the control group you will complete 4 study visits.

If you are in the treatment group you will complete 7 study visits.

The researchers will ask you to answer questionnaires and participate in the management for your pain.

How is this study going to help you?
If you are in the study, you will be helping the researchers answer the study question. To uncover the strengths and weaknesses of the Life Care Specialist position. In addition, the value it provides to the patient and healthcare setting and the resources required to carry through.

Alternatives to Joining This Study

If you decide not to enter this study, there is care available to you outside of this research study. You do not have to be in this study to be treated for your injury.

Costs

There are no costs for your participation on this study.

What Should I Do Next?

Read this form, or have it read to you. Make sure the study doctor or study staff explains the study to you. Ask questions (e.g., about exact time commitment, about unfamiliar words, more details on specific procedures, etc.) Take time to consider this, and talk about it with your family and friends.

Emory University and Grady Health System
Consent to be a Research Subject / HIPAA Authorization

Title: Life Care Specialist (LCS)

Principal Investigator: Dr. Mara Schenker

Sponsor: Christopher Wolf Crusade (CWC)

Investigator-Sponsor: Cammie Wolf Rice

Introduction
You are being asked to be in a medical research study. This form is designed to tell you everything you need to think about before you decide if you want to be a part of the study. **It is entirely your choice. If you decide to take part, you can change your mind later on and withdraw from the research study.** The decision to join or not join the research study will not cause you to lose any medical benefits. If you decide not to take part in this study, your doctor will continue to treat you.

Before making your decision:
- Please carefully read this form or have it read to you
- Please listen to the study doctor or study staff explain the study to you
- Please ask questions about anything that is not clear

You can take a copy of this consent form, to keep. Feel free to take your time thinking about whether you would like to participate. You may wish to discuss your decision with family or friends. Do not sign this consent form unless you have had a chance to ask questions and get answers that make sense to you. By signing this form you will not give up any legal rights.

A description of this clinical trial will be available on http://www.ClinicalTrials.gov, as required by U.S. law. This Web site will not include information that can identify you. At most the Web site will include a summary of the results. You can search this Web site at any time.

What is the purpose of this study?
The main focus of the Christopher Wolf Crusade (CWC) is prevention through the use of a Life Care Specialist (LCS). We are working with key stakeholders in the opioid epidemic to develop an official pain management protocol, as well as conducting an introductory study for a new field of Pain Management.

The goal is to see Life Care Specialists (LCS) staffed in hospitals to focus on pain management and addiction prevention for patients. The LCS position does not currently exist in the healthcare field. Additionally, it will help provide information about the resources that are needed to carry it through.

What will I be asked to do?
If you agree to be a part of the study and are randomized to the control group you will have 4 study visits: hospital admission, 2 week, 6 week and 3 month follow-up.

If you agree to be a part of the study and are randomized to the treatment group you will have 7 study visits: hospital admission, 2 week, 6 week and 3 month follow-up. You'll also complete LCS interventions at your 2 week, 6 week and 3 month follow-ups.

MOD0010-IRB00115061
IRB Approved
4/11/2021

You will be asked to complete questionnaires in person and via text messaging (SMS) that will give us information about your general health and your pain at each appointment with your surgeon. If any questions make you too uncomfortable to answer, you can skip them. Additionally, we will collect information from your hospital admission.

You will be asked to complete the following questionnaires that ask questions about your pain and your health. The questionnaires are:

- Demographics
- Comprehensive social determinants of health survey (SDOH)
- Opioid Risk Tool (ORT)
- Pain Management Questionnaire (PMQ)
- Prescription Drug Use Questionnaire (PDUQ)
- PROMIS: Sleep Disturbance
- PROMIS: Physical Function
- PROMIS: Pain Interference
- Health Misinformation
- Pain Management Survey
- Patient Satisfaction Survey
- Narcan Questionnaire
- PTSD Screener

The following will be collected via text (SMS):

- Actigraphy collects sleep and activity data
- NRS Pain Scores
- Opioid Use

Who owns my study information?
If you join this study, you will be donating your study information. If you withdraw from the study, data that was already collected may be still be used for this study. Study reporting will not identify any one person.

What are the possible risks and discomforts?
There may be side effects from the study or procedures that are not known at this time. Rare but possible risks include: Breach of confidentiality; however, we will be sure to keep all of your protected health information in a password protected, encrypted database only accessible to study team members.

We are asking you to complete questionnaires asking about many social factors that may be sensitive and potentially emotionally upsetting. This includes information about your socioeconomic position, race, ethnic group, cultural context, gender, sexual orientation, alcohol and drug abuse, family and domestic abuse, social relationships, and residential and community context. The questionnaires will be completed in-person, over the phone and/or text messaging (SMS). If any questions make you feel uncomfortable, you can skip them.

Though you will be using pain medications as prescribed, the doctors acknowledge that you are asked to quantify the consumption of a controlled substance, which may be a sensitive matter. Thus, in order to further protect your confidentiality of responses to survey questions, the text messaging (SMS) and online survey responses will be stored as a set of numbers only, without identifying the question to which those numbers pertain. This data will be stored in an encrypted fashion on a commercial cloud server, which is password protected and only accessible by Emory research staff. The survey key linking the questions to the your responses will also only be available to Emory investigators. All protected health information for this study, with the exception of a cell phone number, as it is required to send you text

message survey questions, will be stored separately from the daily survey responses. Your participation in the text message survey may be stopped at any time by responding 'Stop' to a survey question.

Will I benefit directly from the study?
The goal is to introduce a Life Care Specialist (LCS) as an integral member of the clinical team, with a focus on "pain coaching" for trauma patients. The study results may be used to help others in the future.

Will I be compensated for my time and effort?
On your first visit, you will get a gift bag (blanket, bag, water bottle, pen, T-shirt, hat, toothbrush and toothpaste).

At your 2 week and 6 week visit, you will get a vendor-issued gift card, with a cash value of less than $10.

At the conclusion of the study (3 months), you will get a Visa gift card, with a value of $25.

How will you protect my private information that you collect in this study?
Whenever possible, a study number, rather than your name, will be used on study records. Your name and other identifying information will not appear when we present or publish the study results.

Storing and Sharing your Information
De-identified data from this study (data that has been stripped of all information that can identify you), may be placed into public databases where, in addition to having no direct identifiers, researchers will need to sign data use agreements before accessing the data. We will remove or code any personal information that could identify you before your information is shared. This will ensure that, by current scientific standards and known methods, it is extremely unlikely that anyone would be able to identify you from the information we share. Despite these measures, we cannot guarantee anonymity of your personal data.

Your data from this study may be useful for other research being done by investigators at Emory or elsewhere. To help further science, we may provide your de-identified data to other researchers. If we do, we will not include any information that could identify you. If your data is labeled with your study ID, we will not allow the other investigators to link that ID to your identifiable information.

In general, we will not give you any individual results from the study of the data you give us. If we find something of urgent medical importance to you, we will inform you, although we expect that this will be a very rare occurrence.

Medical Record
If you have been an Emory and Grady Health System patient before, then you already have an Emory and Grady Health System medical record. If you have never been an Emory and Grady Health System patient, you do not have one. An Emory and Grady Health System medical record will be made for you if an Emory and Grady Health System provider or facility gives you any services or procedures for this study.

Copies of the consent form/HIPAA authorization that you sign will be put in any Emory and Grady Health System medical record you have now or any time during the study.

Tests and procedures done at non-Emory and Grady Health System places may not become part of your Emory and Grady Health System medical record. Also, if you decide to be in this study, it is up to you to let your other health providers know.

Costs
There will be no costs to you for participating in this study, other than basic expenses like transportation. You will not be charged for any of the research activities.

Withdrawal from the Study
You have the right to leave a study at any time without penalty.

The researchers also have the right to stop your participation in this study without your consent for any reason, especially if they believe it is in your best interest or if you were to object to any future changes that may be made in the study plan.

Authorization to Use and Disclose Protected Health Information

The privacy of your health information is important to us. We call your health information that identifies you, your "protected health information" or "PHI." To protect your PHI, we will follow federal and state privacy laws, including the Health Insurance Portability and Accountability Act and regulations (HIPAA). We refer to all of these laws as the "Privacy Rules." Here we let you know how we will use and disclose your PHI for the study.

PHI that Will be Used/Disclosed:
The PHI that we will use or share for the main research study includes:

- Medical information about you including your medical history and present/past medications.
- Results of exams, procedures and tests you have before and during the study.
- Information related to your mental health.
- Information related to your pain management.

Purposes for Which Your PHI Will be Used/Disclosed:
We will use and share your PHI for the conduct and oversight of the research study. We will use and share your PHI to provide you with study related treatment and for payment for such treatment. We will also use and share your PHI to conduct normal business operations. We may share your PHI with other people and places that help us conduct or carry out the study, such as laboratories, data management centers, data monitors, contract research organizations, Institutional Review Boards (IRBs) and other study sites. If you leave the study, we may use your PHI to determine your health, vital status or contact information.

Use and Disclosure of Your Information That is Required by Law:
We will use and disclose your PHI when we are required to do so by law. This includes laws that require us to report child abuse or abuse of elderly or disabled adults. We will also comply with legal requests or orders that require us to disclose your PHI. These include subpoenas or court orders.

Authorization to Use PHI is Required to Participate:
By signing this form, you give us permission to use and share your PHI as described in this document. You do not have to sign this form. If you do not sign this form, you may still receive non-research related treatment.

People Who will Use/Disclose Your PHI:
The following people and groups will use and disclose your PHI in connection with the research study:

- The Principal Investigator and the research staff will use and disclose your PHI to conduct the study and give you study related treatment.
- The Principal Investigator and research staff will share your PHI with other people and groups to help conduct the study or to provide oversight for the study.

- Christopher Wolf Crusade (CWC) is the Sponsor of the study. The Sponsor may use and disclose your PHI to make sure the research is done correctly and to collect and analyze the results of the research. The Sponsor may disclose your PHI to other people and groups like study monitors to help conduct the study or to provide oversight for the study.
- The following people and groups will use your PHI to make sure the research is done correctly and safely:
    - Emory and Grady Health System offices that are part of the Human Research Participant Protection Program and those that are involved in study administration and billing. These include the Emory IRB, the Grady Research Oversight Committee, the Emory Research and Healthcare Compliance Offices, and the Emory Office for Clinical Research.
    - Public health agencies.
    - Research monitors and reviewer.
    - Accreditation agencies.

Expiration of Your Authorization
Your PHI will be used until this research study ends.

Revoking Your Authorization
If you sign this form, at any time later you may revoke (take back) your permission to use your information. If you want to do this, you must contact Erika Ortega at 404-251-8953.

At that point, the researchers would not collect any more of your PHI. But they may use or disclose the information you already gave them so they can follow the law, protect your safety, or make sure that the study was done properly and the data is correct. If you revoke your authorization you will not be able to stay in the study.

Other Items You Should Know about Your Privacy
Not all people and entities are covered by the Privacy Rules. HIPAA only applies to health care providers, health care payers, and health care clearinghouses. If we disclose your information to people who are not covered by the Privacy Rules, including HIPAA, then your information won't be protected by the Privacy Rules. People who do not have to follow the Privacy rules can use or disclose your information with others without your permission if they are allowed to do so by the laws that cover them. The Sponsor, and people and companies working with the Sponsor on this study are not covered by the Privacy Rules. They will only use and disclose your information as described in this Consent and Authorization.

To maintain the integrity of this research study, you generally will not have access to your PHI related to this research until the study is complete. When the study ends, and at your request, you generally will have access to your PHI that we maintain in a designated record set. A designated record set is data that includes medical information or billing records that your health care providers use to make decisions about you. If it is necessary for your health care, your health information will be provided to your doctor.

We may remove identifying information from your PHI. Once we do this, the remaining information will not be subject to the Privacy Rules. Information without identifiers may be used or disclosed with other people or organizations for purposes besides this study.

<u>Contact Information</u>
Contact Erika Ortega at 404-251-8953:
- if you have any questions about this study or your part in it,
- if you have questions, concerns or complaints about the research

Contact the Emory University Institutional Review Board at 404-712-0720 or 877-503-9797 or irb@emory.edu:

- if you have questions about your rights as a research participant.
- if you have questions, concerns or complaints about the research.
- You may also let the IRB know about your experience as a research participant through our Research Participant Survey at http://www.surveymonkey.com/s/6ZDMW75.

If you are a patient receiving care from the Grady Health System and have a question about your rights, you may contact the Office of Research Administration at research@gmh.edu.

Consent and Authorization

TO BE FILLED OUT BY SUBJECT ONLY

Please **print** your name, **sign**, and **date** below if you agree to be in this research study. By signing this consent and authorization form, you will not give up any of your legal rights. We will give you a copy of the signed form to keep.

____

Patient Name

____ ______________

Patient Signature (18 or older and able to consent) **Date** **Time**

TO BE FILLED OUT BY STUDY TEAM ONLY

____

Name of Person Conducting Informed Consent Discussion

____ ______________

Signature of Person Conducting Informed Consent Discussion **Date** **Time**
